# Altitudinal and Habitat‐Dependent Morphological and Biochemical Variations in *Crepidium acuminatum* (D.Don) Szlach. in the Western Himalaya

**DOI:** 10.1002/cbdv.71662

**Published:** 2026-09-21

**Authors:** Kalpana Rautela, Arun K. Jugran, Amit Bahukhandi, I.D. Bhatt, Prem Prakash

**Affiliations:** ^1^ Garhwal Regional Centre G. B. Pant National Institute of Himalayan Environment Srinagar Uttarakhand India; ^2^ Department of Botany S. B. S. Govt. P. G. College Rudrapur, Udham Singh Nagar Uttarakhand India; ^3^ Centre for Biodiversity Conservation and Management G. B. Pant National Institute of Himalayan Environment Almora Uttarakhand India

**Keywords:** conservation and management, *Crepidium*, IHR, *Malaxis*, medicinal and aromatic plants, morphological studies, phytochemicals, threats

## Abstract

*Crepidium acuminatum* (D.Don) Szlach. is a medicinal orchid used in Ayurvedic formulations, such as Astavarga and Chyawanprash Rasayana. The present study assessed morphological and biochemical variations among diverse populations of *C. acuminatum*. The Thalkedar population exhibited maximum plant height, stem length, leaf length, and pseudobulb length. Similarly, the highest carbohydrate (root part 45.65 ± 1.52%) and flavonoid content (aerial part 2.09 ± 0.26 mg QE/g) were recorded in the Pandukholi population. Maximum protein (aerial part 4.28 ± 0.32%) and phenolic content (root part 2.13 ± 0.15 mg GAE/g) occurred in the Thalkedar population, while the highest methionine content (root part 9.86 ± 0.25%) was observed in the Ramgarh population. Correlation analysis revealed significantly positive relationships (*p* < 0.05) among traits, including pseudobulb length with plant height (*r* = 0.804), and pseudobulb width with stem length (*r* = 0.789). Elevation also showed a strong positive correlation with protein content (*r* = 0.887) and phenolic content in aerial (*r* = 0.873) and root parts (*r* = 0.878). The study identifies particular populations that need conservation and others with commercial potential. It concludes that developing conservation policies and domestication trials is vital for the management of this valuable species.

## Graphical abstract

The study analyzes morphological and biochemical diversity among populations of *Crepidium acuminatum*, a medicinal orchid utilized in Ayurvedic preparations. Distinct populations displayed varied plant traits and metabolite profiles, with altitude positively affecting protein and phenolic content. Findings emphasize populations of conservation importance and those with high commercial value for sustainable use.

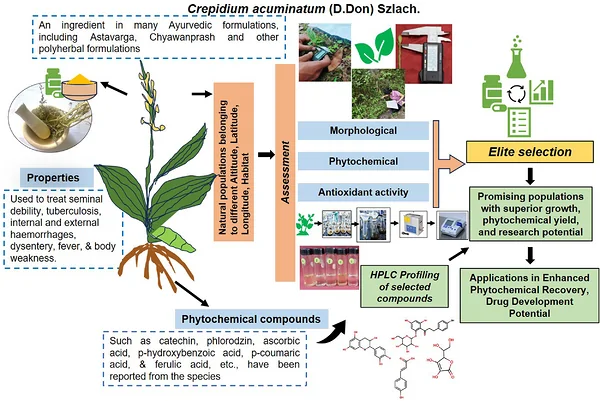

**Abbreviations**
AG: above‐ground (aerial) part
Alt 1: 1500 to 1700 m (*n* = 4)
Alt 2: 1701 to 1900 m (*n* = 12)
Alt 3: 1901 to 2100 m (*n* = 12)
Alt 4: 2101 to 2300 m (*n* = 7)
BG: below‐ground part
CarAG: carbohydrate above‐ground (aerial) part
CarBG: carbohydrate below‐ground part
DpphAG: DPPH‐ 2, 2‐diphenyl‐1‐picryhydrazyl assay in aerial part (mM AAE/100 g DW)
DpphBG: DPPH‐ assay in below‐ground part (mM AAE/100 g DW)
FlavoAG: total flavonoid content in aerial part (mg GAE/g DW)
FlavoBG: total flavonoid content in below‐ground part (mg QE/g DW)
FN: number of flowers per inflorescence
FrapAG: ferric reducing antioxidant power assay in aerial part (mM AAE/100 g DW)
FrapBG: ferric reducing antioxidant power assay in the below‐ground part (mM AAE/100 g DW)
Hab 1: mixed broadleaf forest
Hab 2: cedrus forest
Hab 3: mixed forest
Hab 4: pine‐dominated forest
Hab 5: oak‐dominated forest
LL: lamina length (cm)
LN: number of leaves per plant
LW: lamina width (cm)
MetAG: methionine above‐ground (aerial) part
MetBG: methionine below‐ground part
PBD: pseudobulb diameter (cm)
PBL: pseudobulb length (cm)
PH: plant height (cm)
PheAG: total phenol content in aerial part (mg GAE/g DW)
PheBG: total phenol content in below‐ground part (mg GAE/g DW)
ProAG: protein above‐ground (aerial) part
ProBG: protein below‐ground part
SL: stem length (cm)
SPIKE: spike length (cm)
SW: stem width (cm)
TanAG: total tannin content in aerial part (mg TAE/g DW)
TanBG: total tannin content in aerial part (mg TAE/g DW)
VN: number of veins on the lamina

## 1 Introduction

*Crepidium acuminatum* (D.Don) Szlach (Family Orchidaceae, common name Jeevak, syn. *Malaxis acuminata *D. Don) is an herbaceous terrestrial species, dispersed in Afghanistan, Burma, Bhutan, China, Pakistan, Nepal and the Indo‐China region [1]. The plants are erect and grow up to 30 cm with one flowering axis and a swollen base (pseudobulbous) covered by sheathing leaf bases with 3–5 alternate, broadly lance‐like, acuminate leaves with parallel venation [2]. Pseudobulbs are fleshy, green, long (0.7–9.0 cm), oval or conical shaped with large, elongated rhizomes, yellowish green flowers and ovoid‐shaped fruits [3]. The pseudobulb is the main plant portion of the species used in folk and Ayurvedic medicine, which is commonly used in combination with drugs of other *Astavarga* plant species in Ayurveda [2], and used in polyherbal Ayurvedic formulations, mainly Astavarga, Brahini‐gutika, Chyawanprash rasayana, Himvana agada, Jivaniya‐ghrita [1, 4, 5]. Astavarga is an important group of eight herbaceous plant species, namely Kakoli, Kshirkakoli, Jeevak, Rishbhak, Meda, Mahameda, Riddhi, and Vriddhi [6]. Astavarga and Chyawanprash are commonly consumed to boost immunity and also serve as an energy tonic. The paste of fresh bulbs of *C. acuminatum* is applied to cure phthisis, burning sensation, and fever by the folks of Nagaland, India [7]. The species is highly valued for its diverse medicinal properties and is extensively utilized as an immune modulator, brain tonic, rejuvenating agent, and aphrodisiac. Therapeutically, the species is used in the treatment of bleeding disorders, gastrointestinal issues, insect bites and rheumatism, burning sensations, fever, respiratory disorders, cardiovascular diseases, and internal and external hemorrhage [1, 4, 8, 9]. *C. acuminatum* is also known to possess positive effects for promoting fertility and being used as an antidiarrheal, anti‐inflammatory, febrifuge, and to treat tuberculosis [10, 11]. The species possesses antioxidant, anti‐inflammatory, UV‐A blocking, and revitalizing properties, which support its traditional use. The pseudobulbs of the species contain moisture (6.8%), ash (1.49–6.9% w/w), fat (1.45%), and carbohydrate (112 µg/mL), potassium (21,600 ppm), zinc (43 ppm), magnesium (2800 ppm), manganese (35 ppm), copper (6.48 ppm), calcium (9000 ppm), iron (331 ppm), aluminum (198 ppm), γ‐tocopherol (695.00–786.7 mg/100 g dw) and α‐tocopherol (12.00–9.80 mg/100 dw) [1, 12]. Further, phenolic constituents like catechin, caffeic acid, ferulic acid, phloridzin, chlorogenic acid, ellagic acid, rutin, 3‐hydroxy cinnamic acid, *p*‐coumaric acid, 3‐hydroxybenzoic acid, 4‐hydroxybenzoic acid, and protocatechuic acid, and so forth have also been reported from the species [5, 13].

Therefore, morphological and biochemical characterization and maintenance of elite germplasm will help in the conservation and management of the species. Considering both the economic and conservation significance of the species, the present study was attempted to investigate variations in morphological and phytochemical attributes of different populations of *C. acuminatum* (i) to evaluate the variations in morphological characteristics of *C. acuminatum*; (ii) to investigate the variations in biochemical parameters and antioxidant properties; and, (iii) to prioritize candidate elite germplasm of the species based on the selected morphological and phytochemical traits for conservation and multiplication programmes for the species.

## 2 Materials and Methods

### 2.1 Study Area

Uttarakhand is an important part of the Western Himalayan biogeographic province with diverse habitats, and an altitudinal range forms a broad study area. Thirty‐five distant study sites in Uttarakhand (28°43ˈ to 31°8ˈ N latitude and 77°35ˈto 81°2ˈ E longitude) were identified based on available literature, herbaria records, floras, and reconnaissance surveys, and data and samples were collected from 2022 to 2024 (Figure S1 and Table S1). The herbarium specimen of the species was prepared, identified, and maintained at the BSI herbarium, Dehradun, India (Accession number 1594).

### 2.2 Morphological Assessment

The morphological parameters of ten individuals per population were evaluated across all thirty‐five distant sites of *C. acuminatum* in their natural habitat with minimum soil disturbance. Pseudobulb length and width were recorded by carefully excavating the soil surrounding the plant base. Following measurement, the soil was restored to its original position to minimize disturbance and ensure continued plant survival. The morphological characteristics such as plant height (PH, cm), stem length (SL, cm), stem width (SW, cm), lamina length (LL, cm), lamina width (LW, cm), spike length (SPIKE, cm), pseudobulb length (PBL, cm), pseudobulb diameter (PBD, cm), number of flowers per inflorescence (FN), number of primary branches (PBN), number of spikes (SN), number of leaves per plant (LN), number of veins on the lamina (VN) were measured using a common laboratory scale and Vernier calliper (Mitutoya, Japan) with 0.1 mm accuracy by following standard methods [14, 15]. The characteristics, like the number of primary branches (PBN) and the number of spikes (SN), were excluded from the statistical analysis due to a lack of variation among individuals.

### 2.3 Phytochemical‐Estimation

#### 2.3.1 Extract Preparation

Fresh plant material was brought to the laboratory, washed in tap water followed by distilled water, and divided into two sections (above‐ and below‐ground). The samples were kept in a hot air oven (27°C–30°C) and ground into a fine powder using a mortar and pestle in the laboratory at Garhwal Regional Centre, GBP‐NIHE, Srinagar, Uttarakhand. Precisely, 80% methanol was used for the extraction of dried powder samples (10 mg/mL), macerated in a rotary shaker (160 rpm for 24 h; Remi, India) until decolorized and centrifuged (10 000 rpm for 15 min at 23 ± 1°C), respectively. The mixture was filtered using Whatman filter paper no. 1 and stored in amber‐colored vials (4°C) for the quantification of phytochemical attributes [16]. All the phytochemical assessments were conducted using three biological replicates.

#### 2.3.2 Total Phenolic Contents

Precisely, methanolic extract (0.50 mL) was diluted using distilled water (4.50 mL), Folin–Ciocalteu's reagent (0.50 mL), neutralized the reaction (5.0 mL, 7% sodium carbonate), and kept in the dark (90 min; 25 ± 2°C), respectively, by following Jugran et al. [17]. Absorbance of dark blue color was measured at 765 nm against gallic acid using BioSpectrometer (Eppendorf, Germany). The results of the data were expressed in mg GAE/g dry weight. Phenolic compounds were determined using high‐performance liquid chromatography (HPLC) in GBPNIHE, Kosi, Almora, Uttarakhand.

#### 2.3.3 Total Flavonoid Contents

A mixture of plant extract (1.0 mL) with 10% aluminum chloride (1.0 mL), 1 M potassium acetate (0.20 mL), and distilled water (5.6 mL) was prepared and placed in the dark (30 min; 25 ± 2°C). The absorbance was measured at 415 nm against quercetin using a BioSpectrometer and expressed in mg QE/g dry weight by following Rawat et al. [16].

#### 2.3.4 Total Tannin Contents

Total tannin content was estimated by using the standard methodology [18]. A mixture (extract 2.50 mL, Folin–Dennis reagent 0.25 mL, 7% sodium carbonate 0.50 mL) was prepared, and 3.5 mL of distilled water was added and kept in a water bath (22°C ± 1°C) for 20 min. The green color was measured at 700 nm using a BioSpectrometer against tannic acid and expressed the data in mg TAE/g (mg tannic acid equivalent per gram).

#### 2.3.5 Antioxidant Properties

Two different *in vitro* antioxidant assays, that is, 2,2'‐diphenyl‐1‐picrylhydrazyl (DPPH) radical scavenging assay, and ferric reducing antioxidant power (FRAP) assay were carried out to measure antioxidant properties. Sample extract (1.0 mL) was mixed with 3.0 mL methanolic solution of DPPH reagent and kept in the dark for 20 min (22°C ± 1°C). The purple dark color was measured at 517 nm using BioSpectrometer against ascorbic acid and expressed data in mM AAE/100 g by following Jugran et al. [17]. FRAP reagent (300 mM acetate buffer, pH 3.6, 10 mM 2,4,6‐tri‐2‐pyridyl‐1,3,5‐triazine, 20 mM ferric chloride) was prepared and kept in a water bath (37°C ± 1°C). Then, 0.10 mL of extract and 3.0 mL FRAP reagent were mixed and kept in a water bath (37°C ± 1°C) for 8 min. Absorbance was measured at 593 nm using BioSpectrometer and expressed as data in mM AAE/100 g dry weight.

### 2.4 Nutritional Attributes

#### 2.4.1 Carbohydrate, Protein, and Methionine Estimation

The carbohydrate and protein content were estimated by following Bahukhandi et al. [19] with minor modifications. Briefly, a 50 mg dried powder sample was hydrolyzed with 2.5 mL (2.5 N HCl) and kept in a water bath (100°C ± 1°C; 3 h). After that, the mixture was cooled, neutralized with sodium carbonate, and the makeup volume was 100 mL. The mixture was centrifuged at 5000 rpm (10 min), and the supernatant was collected. Aliquots were mixed with fresh anthrone reagent (0.2% w/v anthrone in 95% H_2_SO_4_), placed in a boiling water bath (100°C ± 1°C; 15 min), and the resulting green‐blue color was measured after cooling at room temperature at 630 nm against a glucose standard using a BioSpectrometer. The results of carbohydrate content were expressed as mg/g dry weight of the sample. Likewise, for protein estimation, a 100 mg sample was homogenized using phosphate‐buffered saline (PBS, pH 7.0), centrifuged at 15 000 rpm (5 min, 4°C). Then, a 10.0 µL aliquot was mixed with Bradford reagent (250 µL) and measured absorbance at 595 nm against BSA using BioSpectrometer, and the results of protein content were expressed as protein %.

The methionine content was estimated by following Jugran et al. [17] with minor changes. 0.5 grams sample was hydrolyzed using 6.0 N HCl and 1.0 gram of activated charcoal, kept in a water bath at 20°C ± 1°C for 10 min. Then, 4.0 mL of deionized water was added, and the filtrate was neutralized with 2.0 mL (5 N NaOH) followed by sequential addition of 0.1 mL (sodium nitroprusside), 2.0 mL (3% glycine solution), 4.0 mL of phosphoric acid, and 2.0 mL (5 N NaOH), respectively. The reaction mixture was acidified with 4.0 mL of phosphoric acid, and color intensity was measured at 450 nm/520 nm using a BioSpectrometer, and the results of methionine content were expressed as % methionine content.

### 2.5 Statistical Analysis

The morphological, phytochemical, antioxidant and nutritional attributes of *C. acuminatum* were carried out in three independent biological replicates. The Duncan's post‐hoc tests (*p*<0.05) were performed using SPSS (version 19.0) to assess the significant differences between the mean values of the analysis and expressed as mean ± standard error (SE). A heatmap was generated to show variations in the studied parameters across the altitudinal gradients and habitat types, where color intensity corresponds to the magnitude of each value: lighter colors indicate lower values, and the darker colors indicate the higher values of each parameter. Pearson correlation coefficients were calculated to evaluate inter‐attribute relationships, followed by neighbor‐joining cluster analysis to identify the major population groups with higher activity and prioritization of the population's elite sites using SPSS software (Version 19.0).

## 3 Results

### 3.1 Morphological Assessment

**FIGURE 1:**
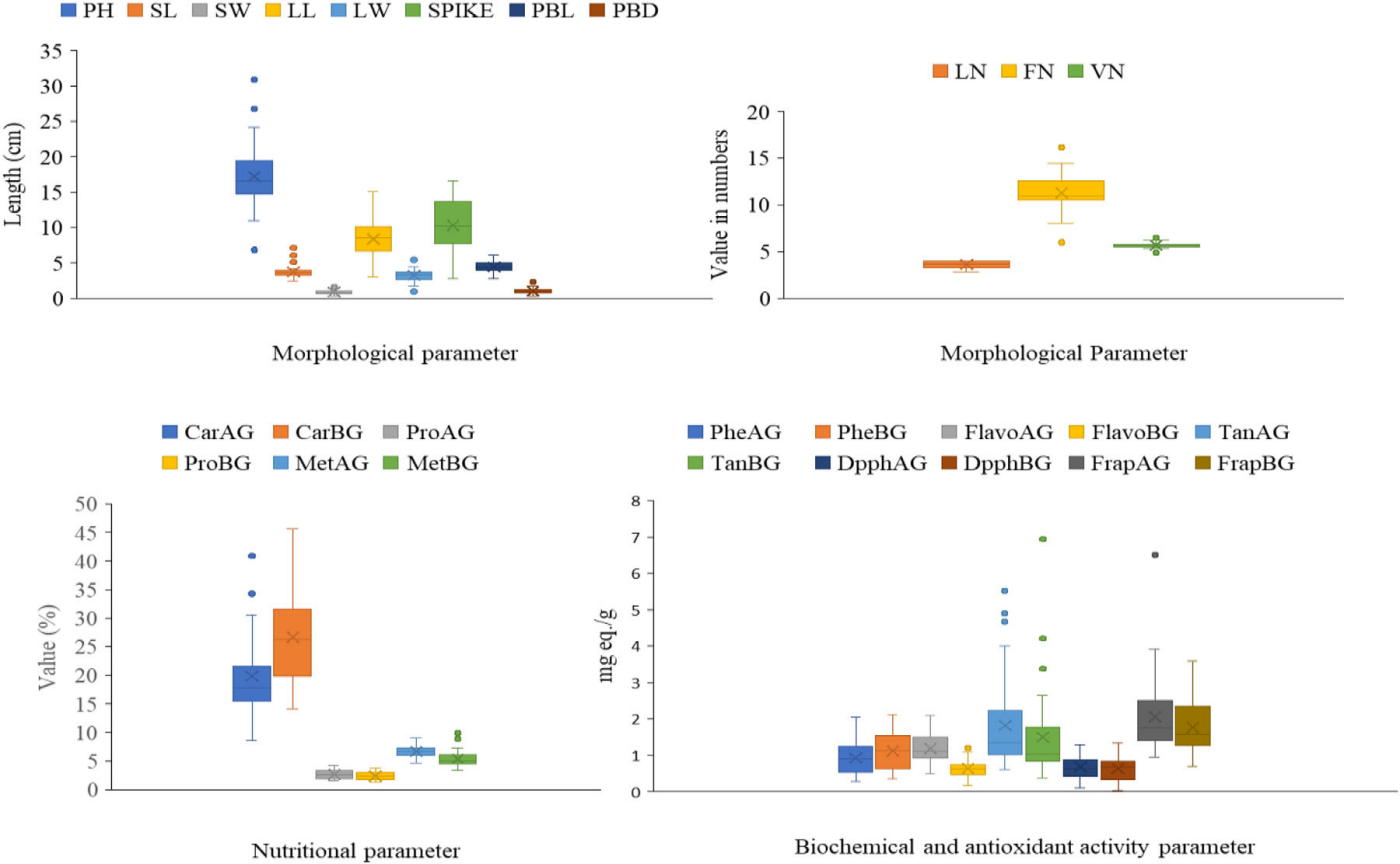
Variations in the morphological, biochemical, phytochemical and antioxidant activity characteristics among the studied populations of *Crepidium acuminatum*. Abbreviations: AG, above ground part; BG, below ground part; Car, carbohydrate; Dpph, DPPH content; Flavo, flavonoid content; FN, number of flowers per inflorescence; Frap, FRAP content; LL, lamina length(cm); LN, number of leaves per plant; LW, lamina width (cm); Met, methionine; PBD, pseudobulb diameter (cm); PBL, pseudobulb length (cm); PH, plant height (cm); Phe, phenolic content; Pro, protein; SL, stem length (cm); SPIKE, spike length (cm); SW, stem width (cm); Tan, tannin content; VN, number of veins on the lamina.

**FIGURE 2:**
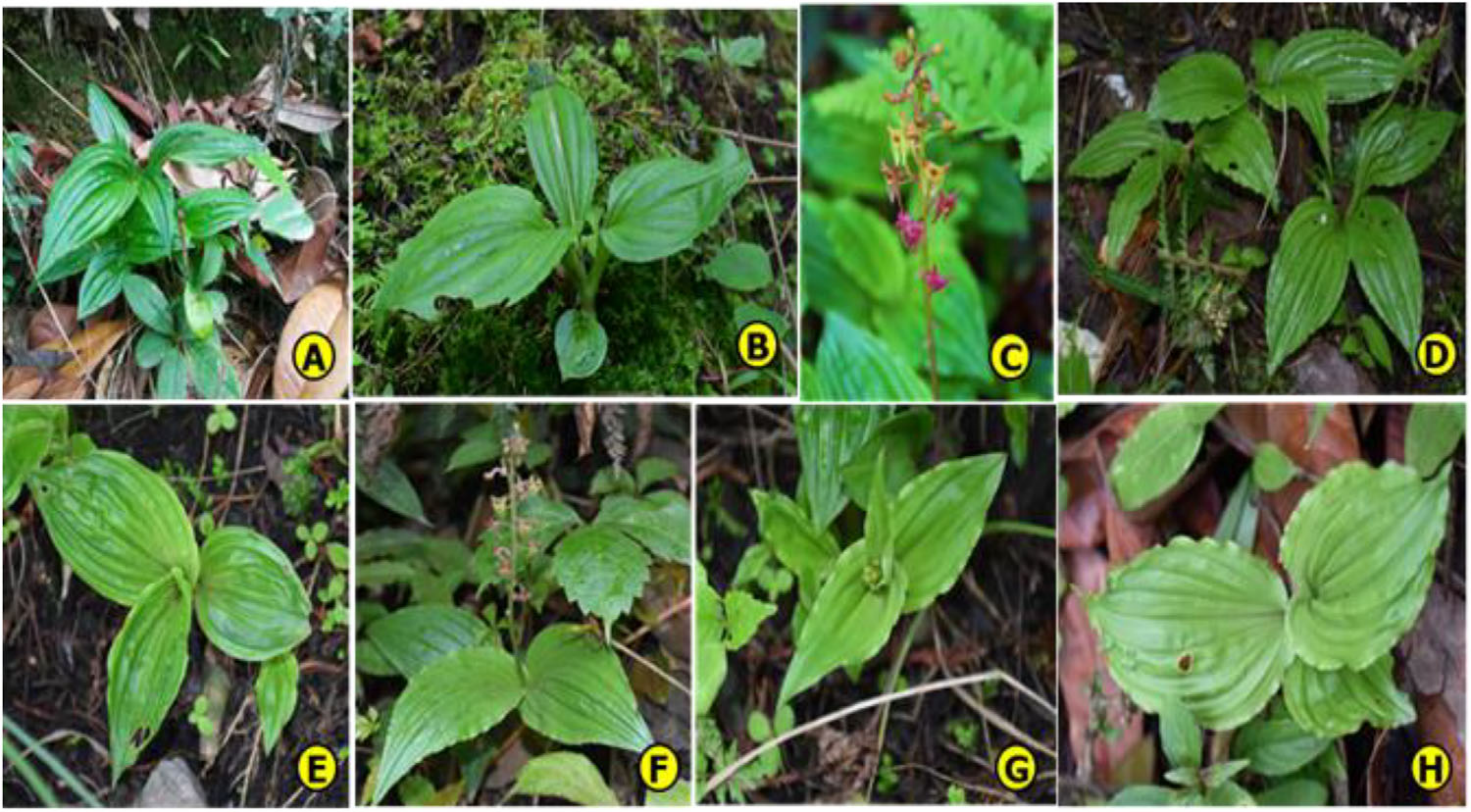
Growth of *Crepidium acuminatum* in different habitat types: (A) Mixed oak forest; (B, C) deodar‐oak forest; (D) deodar forest; (E, F) mixed forest; (G, H) mixed oak‐pine forest.

**FIGURE 3:**
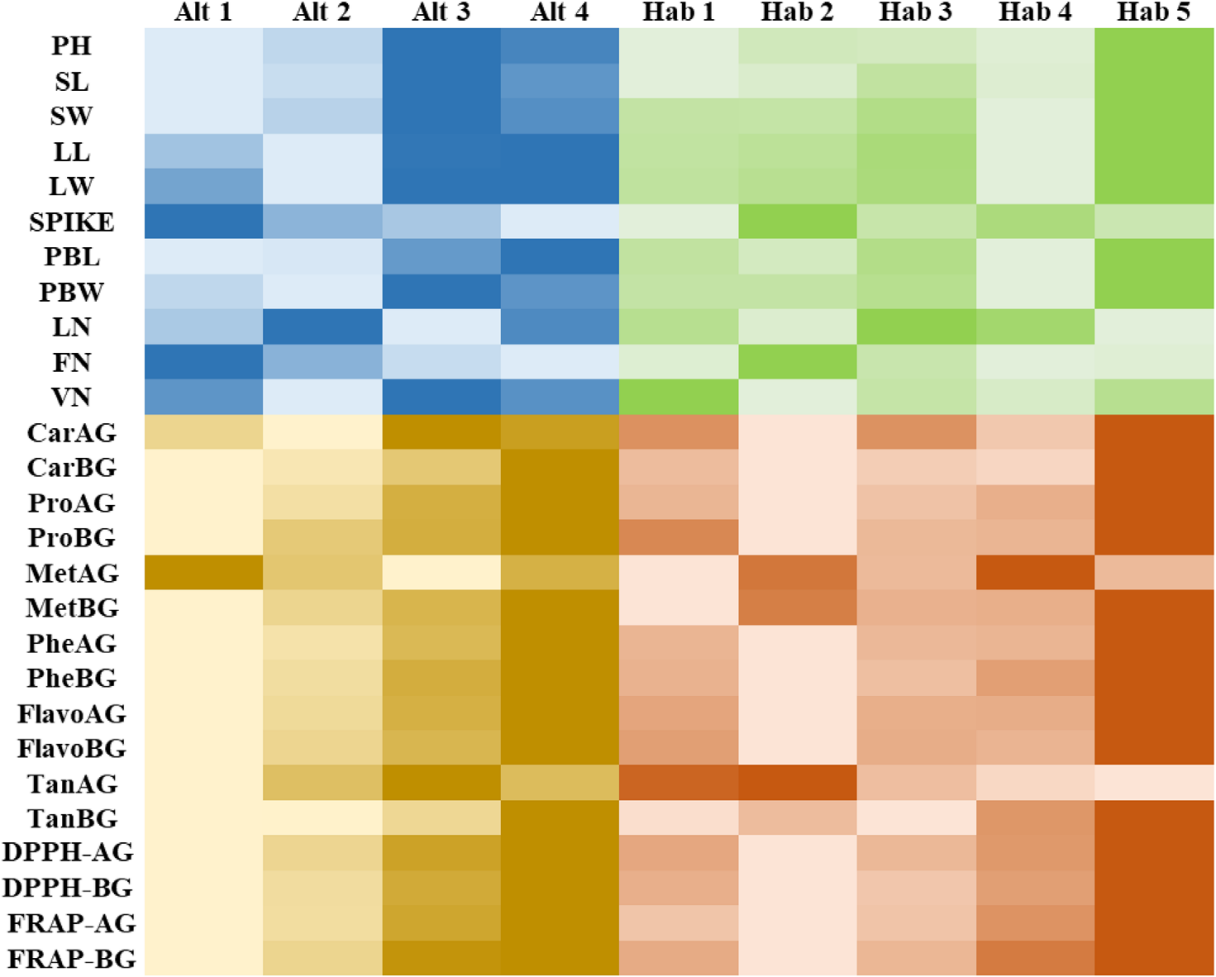
Heatmap illustrating variations in studied morphological, biochemical, phytochemical and antioxidant activity characteristics with a varying range of altitude and habitats, with color intensity representing the magnitude of each value (Lighter color intensity indicates a lower value, and the darker colours indicate the higher values of each parameter). Abbreviations: AG, above ground part; BG, below ground part; Car, carbohydrate; Dpph, DPPH content; Flavo, flavonoid content; FN, number of flowers per inflorescence; Frap, FRAP content; LL, lamina length (cm); LN, number of leaves per plant; LW, lamina width (cm); Met, methionine; PBD, pseudobulb diameter (cm); PBL, pseudobulb length (cm); PH, plant height (cm); Phe, phenolic content; Pro, protein; SL, stem length (cm); SPIKE, spike length (cm); SW, stem width (cm); Tan, tannin content; VN, number of veins on the lamina.

**FIGURE 4:**
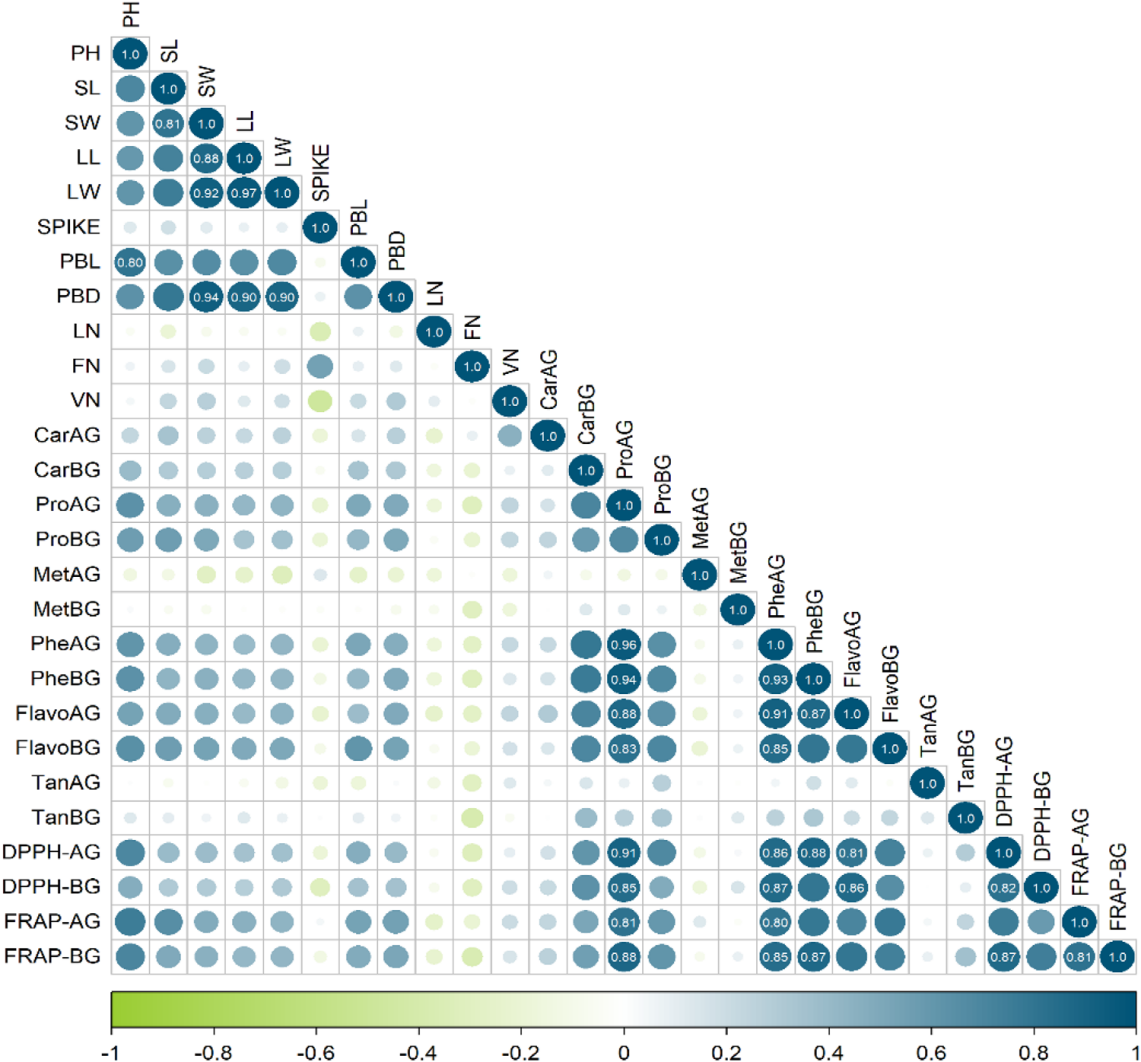
Pearson correlation coefficient analysis among altitude and studied morphological, biochemical, and antioxidant activity parameters of *C. acuminatum*. Abbreviations: AG, above ground part; BG, below ground part; Car, carbohydrate; Dpph, DPPH content; Flavo, flavonoid content; FN, number of flowers per inflorescence; Frap, FRAP content; LL, lamina length (cm); LN, number of leaves per plant; LW, lamina width (cm); Met, methionine; PBD, pseudobulb diameter (cm); PBL, pseudobulb length (cm); PH, plant height (cm); Phe, phenolic content; Pro, protein; SL, stem length (cm); SPIKE, spike length (cm); SW, stem width (cm); Tan, tannin content; VN, number of veins on the lamina.

**FIGURE 5:**
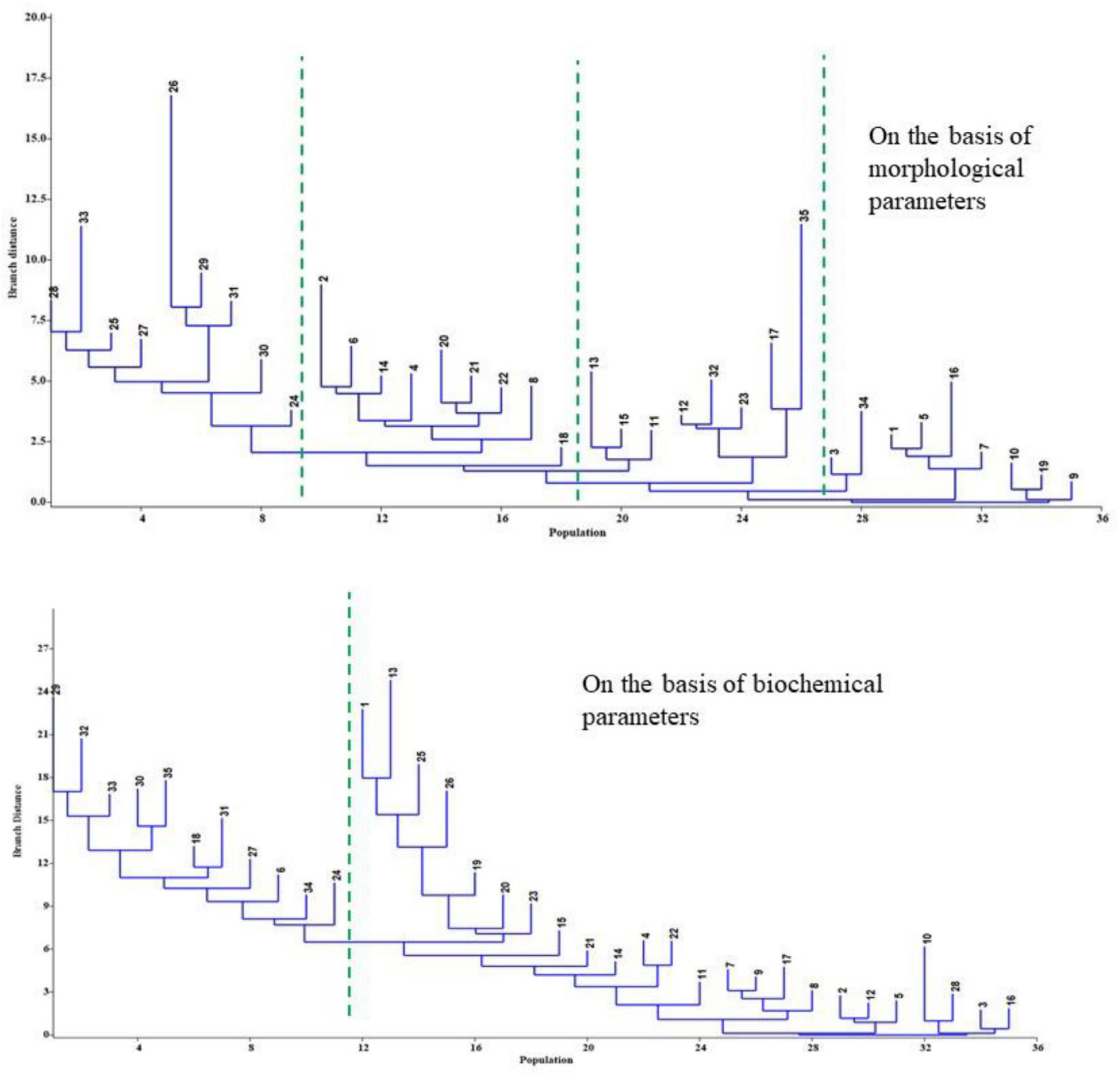
Neighbor‐joining cluster analysis of all the studied biochemical parameters of the *C. acuminatum* populations. *Note*: The numbers denote the populations.

The morphological analysis of quantitative traits across thirty‐five study sites of *C. acuminatum* showed that the population of Thalkedar site exhibited the maximum plant height (PH, 30.93 ± 1.12 cm), stem length (SL, 7.12 ± 0.58 cm), stem width (SW, 1.64 ± 0.03 cm), leaf length (LL, 15.02 ± 0.24 cm) and leaf width (LW, 5.45 ± 0.06 cm) while the Raditop population showed minimum plant height (6.86 ± 0.48 cm) and the Mukteshwar population exhibited minimum SL (2.41 ± 0.16 cm), while the Mallroad population showed the minimum values for SW (0.32 ± 0.11 cm), LL (2.98 ± 0.12 cm), and LW (0.95 ± 0.26 cm) characteristics. Similarly, inflorescence length (SPIKE) and number of flowers (FN) were recorded as maximum (16.64 ± 0.85 cm and 16.2 ± 1.41, respectively) in the Lohaghat population, while the minimum SPIKE length was observed in Jarmola (2.85 ± 0.47 cm). The pseudobulb length (PBL, 6.12 ± 0.21 cm) and pseudobulb diameter (PBD, 2.29 ± 0.07 cm) were found to be maximum in the Thalkedar population, while the minimum PBL (2.82 ± 0.15 cm) and PBD (0.35 ± 0.01 cm) were recorded in the population of Raditop site (Table 1, Figures 1). Similarly, among altitudinal ranges, plant height (19.36 ± 1.28 cm), stem length (3.92 ± 0.35 cm), stem width (0.96 ± 0.11 cm), and leaf width (3.43 ± 0.29 cm) were significantly higher (*p* < 0.05) at 1901–2100 m elevation zone compared to other altitudes, while the minimum plant height (14.25 ± 1.43 cm), stem length (3.52 ± 0.19 cm), stem width (0.82 ± 0.07 cm), and pseudobulb length (4.1875 ± 0.42 cm) were recorded at 1500–1700 m elevation range. Among the habitat types, Oak‐dominated Forest habitats exhibited significantly (*p* < 0.05) higher morphological characteristics, including plant height (21.05 cm), stem length (4.42 cm), stem width (1.13 cm), leaf length (10.06 cm), leaf width (3.94 cm), pseudobulb length (5.18 cm), and pseudobulb width (1.32 cm). In contrast, mixed broadleaf forests showed the lowest plant height (15.18 cm) and stem length (3.26 cm), while pine‐dominated forests recorded the minimum stem width (0.6 cm), leaf length (5.69 cm), leaf width (2.22 cm), pseudobulb length (3.5 cm), and pseudobulb width (0.68 cm) (Figures 2 & 3). Correlation analysis revealed significantly positive relationships (*p* < 0.05) between various morphological traits of *C. acuminatum*, such as leaf length with leaf width (*r* = 0.965), pseudobulb length with plant height (*r* = 0.804), and stem length (*r* = 0.642), pseudobulb width with stem length (*r* = 0.789), stem width (*r* = 0.938), leaf length (*r* = 0.897), leaf width (*r* = 0.899). Additionally, stem width was positively correlated with leaf length (*r* = 0.879) and leaf width (*r* = 0.924) (Figure 4). Neighbor‐joining (NJ) cluster analysis based on morphological characteristics identified four representative clusters of the populations: Cluster I contain Ramgarh, Farsundakhal, Thalkedar, Sirakot forest, Berinag, Pandukholi, Syahidevi, Doonagiri, and Munsiyari and Cluster II includes Lohaghat, Mayawati ashram, Majkhali, Gangolihat, Chandak, Hanumangarhi, Jageshwar, Madhyamaheshwar, and Chaubattia. Similarly, Cluster III contains Dol Ashram, Nagdev forest, Jakholi, Khirshu, Jarmola, Sandev forest, Chakrata, and Raditop, whereas Cluster IV consists of Lweshal, Bhowali, Lamgarha, Kausani, Salyura, Kalika, Mallroad, Devidhura, and Mukteshwar (Figure 5).

**TABLE 1:** Morphological parameters of different populations of *C. acuminatum* collected from Uttarakhand, Western Himalaya.

Population (code)	PH	SL	SW	LL	LW	SPIKE	PBL	PBD	LN	FN	VN
**Lweshal (CA1)**	13.62 ± 1.01^nopqr^	2.99 ± 0.19^ijklmn^	0.63 ± 0.06^ij^	6.13 ± 0.5^jkl^	2.68 ± 0.27^jklm^	12.01 ± 0.88^cdefg^	3.88 ± 0.18^ijklm^	0.69 ± 0.06 ^klm^	3.3 ± 0.21^bcde^	12.6 ± 0.87^bcdef^	6.2 ± 0.33^ab^
**Lohaghat (CA2)**	11.73 ± 0.82^qr^	3.53 ± 0.13^defghijk^	0.79 ± 0.03^fghi^	7.86 ± 0.65^ghij^	3.22 ± 0.16^efghijk^	16.64 ± 0.85^a^	3.16 ± 0.13^klmn^	0.87 ± 0.05^hijkl^	3.4 ± 0.22^abcde^	16.2 ± 1.41^a^	5.3 ± 0.15^cd^
**Bhowali (CA3)**	13.28 ± 0.87^opqr^	3.69 ± 0.28^defghi^	0.92 ± 0.07^efgh^	8.85 ± 0.58^defg^	3.57 ± 0.31^cdefgh^	9.25 ± 0.88^hijkl^	4.74 ± 0.49^bcdefghi^	1.01 ± 0.08^fghij^	3.8 ± 0.13^abc^	10.5 ± 0.95^cdefghi^	5.7 ± 0.21^abcd^
**Mayawati (CA4)**	18.37 ± 1.2^ghij^	3.87 ± 0.38^defgh^	0.96 ± 0.05^defg^	9.92 ± 0.91^cdef^	3.67 ± 0.27^bcdefg^	13.1 ± 1.63^bcd^	4.97 ± 0.48^bcdefgh^	1.26 ± 0.22^cdef^	3.9 ± 0.1^ab^	13.13 ± 1.52^bcd^	5.7 ± 0.26^abcd^
**Lamgarha (CA5)**	14.2 ± 1.1l^mnopq^	2.55 ± 0.14^mn^	0.71 ± 0.05^ghi^	5.59 ± 0.39^kl^	2.41 ± 0.18^klmn^	12.3 ± 0.68^cde^	4.23 ± 0.45^fghij^	0.48 ± 0.04^mn^	4 ± 0^a^	13.5 ± 0.85^bc^	5.6 ± 0.16^bcd^
**Majkhali (CA6)**	16.95 ± 0.68^hijklm^	3.67 ± 0.48^defghi^	0.84 ± 0.04^efghi^	8.77 ± 0.58^efg^	3.51 ± 0.28c^defghi^	15.25 ± 0.79^ab^	4.54 ± 0.44^defghi^	0.96 ± 0.07^ghijk^	3.6 ± 0.27^abcd^	14.5 ± 0.6^ab^	5.6 ± 0.16^bcd^
**Kausani (CA7)**	13.85 ± 0.84^mnopqr^	2.85 ± 0.13^jklmn^	0.69 ± 0.04^hi^	5.79 ± 0.18^kl^	2.59 ± 0.12^jklm^	10.5 ± 1.12^efghi^	3.37 ± 0.19^jklmn^	0.69 ± 0.06^klm^	3.4 ± 0.31^abcde^	11 ± 1.36cd^efgh^	5.4 ± 0.16^bcd^
**Gangolihat (CA8)**	14.9 ± 1.32^lmnop^	3.62 ± 0.14^defghij^	0.82 ± 0.06^fghi^	10.68 ± 0.32^kl^	3.37 ± 0.21^jklm^	13.81 ± 0.51^bc^	4.06 ± 0.23^hijkl^	0.94 ± 0.08^efghi^	3.4 ± 0.3^bcde^	10.5 ± 0.82^cdefghi^	4.9 ± 0.41^d^
**Salyura (CA9)**	14.94 ± 0.78^lmnop^	3.34 ± 0.2^efghijkl^	0.77 ± 0.04^ghi^	7.72 ± 0.8^ghij^	2.85 ± 0.24^hijklm^	9.79 ± 0.63^fghijkl^	4.38 ± 0.16^efghi^	0.78 ± 0.08^ijkl^	4 ± 0^a^	10.14 ± 1.05^defghi^	5.7 ± 0.21^abcd^
**Kalika (CA10)**	14.84 ± 0.99^lmnop^	3.28 ± 0.44^fghijklm^	0.75 ± 0.07^ghi^	6.75 ± 0.38^hijk^	2.77 ± 0.14^jklm^	9.2 ± 0.53^hijkl^	4 ± 0.3^hijkl^	0.77 ± 0.05^jklm^	4 ± 0^a^	8 ± 0.58^ij^	5.4 ± 0.16^bcd^
**Dol Ashram (CA11)**	16.63 ± 0.65^hijklmn^	3.98 ± 0.19^defgh^	1.04 ± 0.07^def^	10.1 ± 0.73^cdef^	3.77 ± 0.36^bcdef^	9.23 ± 0.51^hijkl^	4.13 ± 0.21^ghijk^	1.3 ± 0.09d^efgh^	4 ± 0^a^	11.29 ± 0.99^cdefgh^	5.5 ± 0.22^bcd^
**Nagdev forest (CA12)**	17.19 ± 0.83^hijkl^	3.71 ± 0.19^defghi^	0.93 ± 0.12^efgh^	9.16 ± 0.71^cdefg^	3.59 ± 0.23cd^efgh^	6.17 ± 0.17^mn^	5.07 ± 0.19^bcdefg^	1.03 ± 0.05^fghij^	4 ± 0^a^	10.7 ± 0.75c^defghi^	5.7 ± 0.21^abcd^
**Jakholi (CA13)**	15.03 ± 0.54^lmnop^	5.57 ± 0.1^bc^	1.5 ± 0.07^ab^	10.85 ± 0.46^bc^	4.4 ± 0.18^b^	7.38 ± 0.55^lmn^	4.22 ± 0.16^fghij^	1.7 ± 0.09^b^	4 ± 0^a^	14.37 ± 0.44^ab^	6.5 ± 0.31^a^
**Chandak (CA14)**	15.12 ± 0.69^klmnop^	3.48 ± 0.13^defghijk^	0.97 ± 0.05^defg^	7.75 ± 0.42^ghij^	2.98 ± 0.12^fghijkl^	14.13 ± 0.46^bc^	4.79 ± 0.08^bcdefghi^	0.85 ± 0.04^hijkl^	3.2 ± 0.33^cde^	13.5 ± 0.87^bc^	5.5 ± 0.37^bcd^
**Khirshu (CA15)**	15.12 ± 0.8^klmnop^	3.59 ± 0.25^defghij^	0.85 ± 0.1^efghi^	8.78 ± 0.47^efg^	3.55 ± 0.19cd^efgh^	7.75 ± 0.42^jklm^	4.67 ± 0.15c^defghi^	0.96 ± 0.1^ghijk^	3.8 ± 0.13^abc^	12.14 ± 0.98^bcdefgh^	6.2 ± 0.25^ab^
**Mallroad (CA16)**	13.04 ± 0.57^pqr^	3.22 ± 0.23^ghijklm^	0.32 ± 0.11^ghi^	2.98 ± 0.12^m^	0.95 ± 0.26°	12.15 ± 1.05^cdef^	3.11 ± 0.23^lmn^	0.6 ± 0.11^lm^	3.2 ± 0.29^cde^	11 ± 0.65^cdefgh^	5.6 ± 0.16^bcd^
**Jarmola (CA17)**	15.82 ± 1.4^jklmnop^	2.45 ± 0.26^n^	0.44 ± 0.02^j^	4.89 ± 0.19^l^	2.14 ± 0.24^mn^	2.85 ± 0.47°	4.81 ± 0.16^bcdefghi^	0.5 ± 0.04^lmn^	4 ± 0^a^	10.8 ± 0.55^cdefghi^	6.2 ± 0.33^ab^
**Hanumangarhi (CA18)**	16.29 ± 0.89^ijklmno^	3.61 ± 0.2^defghij^	0.816 ± 0.04^fghi^	8.43 ± 0.72^fgh^	3.33 ± 0.44^defghij^	10.33 ± 0.76^efghij^	4.37 ± 0.25^efghi^	0.92 ± 0.11^ghijk^	3.4 ± 0.31^abcde^	11 ± 0.67cd^efgh^	5.4 ± 0.16^bcd^
**Devidhura (CA19)**	14.7 ± 0.5^8lmnop^	3.16 ± 0.24^hijklmn^	0.71 ± 0.03^ghi^	6.67 ± 0.46^ijk^	2.72 ± 0.18^ijklm^	10.25 ± 0.79^efghij^	3.97 ± 0.25^hijkl^	0.75 ± 0.03^jklm^	3.6 ± 0.22^abcd^	9.25 ± 0.7^ghi^	5.6 ± 0.16^bcd^
**Jageshwar (CA20)**	19.42 ± 1.09^fgh^	2.74 ± 0.13^klmn^	0.65 ± 0.06^ij^	5.63 ± 0.32^kl^	2.49 ± 0.2^klm^	13.66 ± 0.81^bc^	3.32 ± 0.14^jklmn^	0.66 ± 0.08^lm^	3.3 ± 0.26^bcde^	12.29 ± 1.06^bcdefg^	5.6 ± 0.22^bcd^
**Madhyamaheshwar (CA21)**	17.15 ± 1.29^hijkl^	3.7 ± 0.22^defghi^	0.59 ± 0.05^ij^	5.56 ± 0.38^kl^	2.37 ± 0.13^lmn^	13.34 ± 1.36^bc^	2.86 ± 0.13^n^	0.6 ± 0.06^lm^	4 ± 0^a^	10.3 ± 0.84^defghi^	5.4 ± 0.16^bcd^
**Chaubattia (CA22)**	16.97 ± 0.5^hijklm^	3.29 ± 0.1^fghijklm^	0.76 ± 0.07^ghi^	7.55 ± 0.71^ghij^	2.84 ± 0.23^hijklm^	13.98 ± 0.53^bc^	4.05 ± 0.26^hijkl^	0.77 ± 0.06^jklm^	4 ± 0^a^	11.75 ± 0.78^bcdefgh^	5.7 ± 0.15^abcd^
**Sandev forest (CA23)**	17.32 ± 0.64^hijkl^	3.75 ± 0.08^defghi^	0.8 ± 0.03^fghi^	8.33 ± 0.12^fghi^	3.32 ± 0.16^defghij^	5.71 ± 0.28^mn^	4.27 ± 0.09^fghij^	0.9 ± 0.07^fghij^	3.2 ± 0.33^cde^	10 ± 0.62^efghi^	5.7 ± 0.37^abcd^
**Ramgarh (CA24)**	18.17 ± 1.85^ghijk^	3.86 ± 0.35^defgh^	0.96 ± 0.04^defg^	9.35 ± 0.78^cdefg^	3.62 ± 0.22^cdefgh^	10.8 ± 1.39^defgh^	4.93 ± 0.92^bcdefgh^	1.23 ± 0.09^cdefg^	3.2 ± 0.25^cde^	10.5 ± 1.06^cdefghi^	5.7 ± 0.15^abcd^
**Farsundakhal (CA25)**	21.74 ± 1.19^cdef^	4.12 ± 0.18^de^	1.19 ± 0.1^cd^	10.58 ± 0.34^bcd^	4.14 ± 0.27^bcd^	8.08 ± 0.58^ijklm^	5.28 ± 0.11^abcde^	1.38 ± 0.16^cd^	3.5 ± 0.17^abcd^	10.7 ± 0.9c^defghi^	5.7 ± 0.21^abcd^
**Thalkedar (CA26)**	30.93 ± 1.12^a^	7.12 ± 0.58^a^	1.64 ± 0.03^a^	15.02 ± 0.24^a^	5.45 ± 0.06^a^	14.43 ± 1.05^abc^	6.12 ± 0.21^a^	2.29 ± 0.07^a^	2.8 ± 0.29^e^	9.7 ± 0.62^fghi^	6 ± 0.26^abc^
**Sirakot (CA27)**	20.69 ± 0.61d^efg^	4.08 ± 0.05^def^	1.61 ± 0.03^a^	11.77 ± 0.4^b^	4.45 ± 0.11^b^	8.91 ± 0.34^hijkl^	5.2 ± 0.13^abcdef^	2.29 ± 0.1^a^	3.6 ± 0.22^abcd^	11.1 ± 0.46^cdefgh^	6.2 ± 0.29^ab^
**Berinag (CA28)**	23.16 ± 0.72^cd^	5.12 ± 0.14^c^	1.34 ± 0.09^bc^	10.68 ± 0.62^bc^	4.31 ± 0.17^bc^	7.63 ± 0.48^klm^	5.61 ± 0.12^abc^	1.45 ± 0.08^c^	4 ± 0^a^	11.8 ± 0.87^bcdefgh^	5.9 ± 0.41^abc^
**Pandukholi (CA29)**	24.16 ± 1.11^c^	6.11 ± 0.1^b^	1.32 ± 0.05^bc^	8.61 ± 0.64^efg^	3.95 ± 0.3^bcde^	13.93 ± 0.71^bc^	6.08 ± 0.43^a^	1.36 ± 0.07^cd^	3.1 ± 0.28^de^	13.14 ± 0.82^bcd^	5.7 ± 0.21^abcd^
**Syahidevi (CA30)**	20.28 ± 0.7^efg^	4 ± 0.2^defg^	1.08 ± 0.11^de^	10.35 ± 0.73^bcde^	3.95 ± 0.35^bcde^	10.11 ± 0.92^efghijk^	5.16 ± 0.24^abcdef^	1.33 ± 0.08^cde^	4 ± 0^a^	9.12 ± 0.48^hi^	5.7 ± 0.21^abcd^
**Doonagiri (CA31)**	22.79 ± 0.52^cde^	4.22 ± 0.15^d^	1.18 ± 0.21^cd^	10.37 ± 0.22^bcde^	4.26 ± 0.32^bc^	13.86 ± 0.52^bc^	5.42 ± 0.07^abcd^	1.43 ± 0.04^c^	4 ± 0^a^	13 ± 0.9^bcde^	5.6 ± 0.22^bcd^
**Chakrata (CA32)**	18.96 ± 1.13^fghi^	3.9 ± 0.11^defgh^	0.78 ± 0.12^fghi^	9.93 ± 0.53^cdef^	3.69 ± 0.22^bcdef^	5.09 ± 0.22^no^	4.98 ± 0.25^bcdefgh^	1.27 ± 0.16^cdefg^	3.9 ± 0.1^ab^	10.5 ± 0.56c^defghi^	5.8 ± 0.29^abc^
**Munsiyari (CA33)**	26.79 ± 0.5^b^	3.37 ± 0.15^efghijkl^	0.78 ± 0.1f^ghi^	7.74 ± 0.18^ghij^	2.87 ± 0.13^ghijklm^	5.98 ± 0.29^mn^	5.73 ± 0.13^ab^	0.8 ± 0.05^hijkl^	4 ± 0^a^	10.6 ± 0.7c^defghi^	5.5 ± 0.22^bcd^
**Mukteshwar (CA34)**	10.95 ± 0.65^r^	2.41 ± 0.16^n^	0.94 ± 0.1d^efgh^	9.32 ± 0.98^cdefg^	3.59 ± 0.43^cdefgh^	9.6 ± 0.87^ghijkl^	2.96 ± 0.49^mn^	1.07 ± 0.09^efghi^	3.2 ± 0.29^cde^	10.5 ± 0.89^cdefghi^	5.6 ± 0.16^bcd^
**Raditop (CA35)**	6.86 ± 0.48^s^	2.68 ± 0.27^lmn^	0.44 ± 0.03^j^	4.72 ± 0.12^l^	1.71 ± 0.24^n^	2.92 ± 0.56°	2.82 ± 0.15^n^	0.35 ± 0.01^n^	3.6 ± 0.22^abcd^	6 ± 0.78^j^	6.2 ± 0.25^ab^

*Note*: Values are presented as means ± standard error (SE). Within columns, SE values followed by different superscript letters (a, b, c, etc.) indicate significant differences (*p*<0.05) according to One‐way ANOVA followed by Duncan multiple range test (DMRT).

Abbreviations: FN, number of flowers per inflorescence; LL, lamina length (cm); LN, number of leaves per plant; LW, lamina width (cm); PBD, pseudobulb diameter (cm); PBL, pseudobulb length (cm); PH, plant height (cm); SL, stem length (cm); SPIKE, spike length (cm); SW, stem width (cm); VN, number of veins on the lamina.

### 3.2 Nutritional Assessment

Significantly higher variations (*p* < 0.05) in the nutritional parameters of *C. acuminatum* populations were recorded using ANOVA analysis (one‐way ANOVA). The plants collected from the Pandukholi site showed the maximum carbohydrate content (45.65 ± 1.52%) in the root parts (BG), while the Kalika population recorded the lowest (8.60 ± 0.82%) carbohydrate content in the aerial parts. The Thalkedar population showed the maximum protein content (4.28 ± 0.32%) in the aerial part, and the Lohaghat population exhibited the lowest protein content (1.27 ± 0.04%) in the root parts. However, the methionine content was found to be maximum in the root parts of Ramgarh (9.86 ± 0.25%). Whereas the minimum methionine content (3.31 ± 0.31%) was found in the root parts of Sirakot (Figures 1; Table S2). Similarly, for variations in altitudinal range, populations at altitude between 2101 and 2300 m had significantly higher carbohydrate content in the root parts (37.71%), protein content in the aerial parts (3.58%) and root parts (3.02%), and methionine content in the root parts (5.54 %). Populations at 1901–2100 m showed a significantly higher carbohydrate content in the aerial part (20.84%). Whereas the populations at the altitude between 1500 and 1700 m recorded the lowest carbohydrate content in the root parts (19.6%), protein content in the aerial (1.7%) as well as in the root (1.52%) parts, and the methionine content in the root (5.14%) parts. Among the habitat types, the oak‐dominated forest habitats recorded significantly higher carbohydrate content in the aerial part (21.73%) as well as in the root (31.14%) parts, protein content in the aerial (3.44%) as well as in the root (2.88%) parts, and the methionine content in the root (6.18%) parts. Whereas pine‐dominated forest habitats recorded the methionine content in the aerial parts (7.33%). Similarly, the populations in the cedrus forest habitat recorded the lowest values for the carbohydrate content in aerial part (16.93 ± 2.43%) as well as in the root parts (23.82 ± 3.67%), protein content in aerial part (1.98 ± 0.31%) as well as in root parts (1.8 ± 0.32%) while the methionine content in the aerial part (6.29 ± 0.26%) and the root parts (4.58 ± 0.41%) was found lowest in the populations of mixed broadleaf forest habitats (Figure 3).

### 3.3 Phytochemical Assessment

The total phenolic content was highest (2.13 ± 0.15 mg GAE/g) in the root part of the Thalkedar population and lowest (0.28 ± 0.02 mg GAE/g) in the aerial part of the Lohaghat population. Similarly, the flavonoid content was reported to be maximum in the aerial part of the Pandukholi population (2.09 ± 0.26 mg QE/g) and minimum in the root parts of the Lweshal population (0.17 ± 0.03 mg QE/g). In the same context, the tannin content was found to be highest in the root parts of the Syahi Devi (6.95 ± 0.08 mg TAE/g) population and lowest (0.38 ± 0.05 mg TAE/g) in the root part of the Mallroad population (Figure 1). Altitude‐wise comparative assessment showed that populations at altitudes between 2101‐2300 m showed a significantly higher (*p* < 0.05) phenolic content in both aerial (1.59 ± 0.1 mg GAE/g) and root parts (1.79 ± 0.07 mg GAE/g), flavonoid content in aerial (1.67 ± 0.1 mg QE/g) and root parts (0.9 ± 0.07 mg QE/g), and tannin content in root parts (2.36 ± 0.84 mg TAE/g) whereas the populations of 1500–1700 m range recorded the lowest values for almost all the phytochemical attributes. Among the habitat types, oak‐dominated forest habitats recorded a significantly higher (*p* < 0.05) values for phenolic content in aerial (1.37 ± 0.15 mg GAE/g) and root parts (1.58 ± 0.18 mg GAE/g), flavonoid content in aerial (1.54 ± 0.14 mg QE/g) and root parts (0.82 ± 0.09 mg QE/g), and tannin content in root parts (2.03 ± 0.65 mg TAE/g). Whereas, cedrus forest habitats demonstrated the lowest phenolic and flavonoid contents, and mixed forest habitats had the minimum tannin content in the root parts (1.21 ± 0.19 mg TAE/g) (Figure 3; Table S2).

### 3.4 Antioxidant Activity Assessment

The antioxidant activity via DPPH assay was found to be maximum in the root parts of the Pandukholi population (1.33 ± 0.09 mM AAE/100 g DW), while it was found to be lowest (0.02 ± 0.01 mM AAE/100 g DW) in the root parts of the Kausani population. Similarly, the FRAP activity was recorded as the highest (6.51 ± 0.3 mM AAE/100 g DW) in the aerial parts of the Thalkedar population and the lowest (0.71 ± 0.05 mM AAE/100 g DW) in the root parts of the Mayawati population (Figure 1). Altitude‐wise comparative assessment revealed that the populations at elevation zone 2101–2300 m demonstrated significantly (*p* < 0.05) higher antioxidant activity via both the assays, DPPH activity in the aerial (1.04 ± 0.08 mM AAE/100 g DW) and root (1.04 ± 0.1 mM AAE/100 g DW) parts and also FRAP activity in aerial (2.89 ± 0.27 mM AAE/100 g DW) as well as in root (2.28 ± 0.14 mM AAE/100 g DW) parts. Whereas populations at the elevation zone of 1500–1700 m showed the least antioxidant activity using both DPPH and FRAP. Likewise, populations in oak‐dominated forest habitats documented significantly (*p* < 0.05) higher antioxidant activity using both DPPH activity in aerial (1.01 ± 0.13 mM AAE/100 g DW) and root (0.95 ± 0.13 mM AAE/100 g DW) parts and also FRAP activity in aerial (2.98 ± 0.52 mM AAE/100 g DW) as well as in the root (2.33 ± 0.23 mM AAE/100 g DW) parts. Whereas the populations residing in the cedrus forest habitat recorded the lowest value for all the antioxidant assays (Figure 3; Table S2).

The study reported a significant positive relationship of elevation with protein content of aerial part (*r* = 0.887), with phenolic content of aerial part (*r* = 0.873) as well as of root parts (*r* = 0.878), with flavonoid content of aerial part (*r* = 0.831), with DPPH activity of aerial (*r* = 0.817) and root (*r* = 0.83) parts. Similarly, protein content of aerial part also recorded positive relation with phenolic content of aerial (*r* = 0.958) and root (*r* = 0.943) parts, with flavonoid content of aerial (*r* = 0.876) and root (*r* = 0.826) parts, with tannin content of aerial part (*r* = 0.85), with DPPH activity of aerial (*r* = 0.90) and root parts (*r* = 0.853) and also with FRAP activity of root (*r* = 0.88) parts. Likewise, phenolic content of aerial part demonstrated a significantly positive relationship with phenolic content of root parts (*r* = 0.92), with flavonoid of aerial (*r* = 0.910) and root parts (*r* = 0.854) part, with DPPH activity of aerial (*r* = 0.86) and of root parts (*r* = 0.86) and also with FRAP activity of aerial (*r* = 0.801) and root parts (*r* = 0.848) (Figure 4). Neighbor‐joining cluster analysis was utilized to identify and prioritize potential populations based on their nutritional, phytochemical, and antioxidant activity attributes, which identified three representative clusters. Cluster I included populations of Pandukholi, Munsiyari, Syahidevi, Chakrata, Raditop, Doonagiri, Sirakot, Hanumangarhi, Majkhali, Mukteshwar, and Ramgarh, while Cluster II comprised the rest of the studied populations (Figure 5).

### 3.5 Quantification of Phenolic Compounds Through HPLC Analysis

HPLC analysis revealed significant variations (*p* < 0.05) in selected polyphenolic compounds of the aerial and root parts among different populations of *C. acuminatum*. In the above‐ground part, various phenolic compounds such as catechin (1.29–44.50 mg/100 g), phlorodzin (3.35–451.40 mg/100 g), ascorbic acid (6.23–80.23 mg/100 g), naringin (1.74–74.06 mg/100 g), *p*‐hydroxy benzoic acid (1.21–19.02 mg/100 g), *o*‐coumaric acid (0.67–7.83 mg/100 g), *p*‐coumaric acid (0.57–8.50 mg/100 g), ferulic acid (0.006–9.91 mg/100 g), epigallocatechin‐3‐gallate (1.82–41.20 mg/100 g), *m*‐coumaric acid (3.50–9.55 mg/100 g), gallic acid (1.33–10.80 mg/100 g) and caffeic acid (0.02–8.38 mg/100 g), and so forth, were reported among the populations of *C. acuminatum*. Whereas, in the below‐ground part, a considerably lower content of these phenolic compounds was reported such as catechin (8.83–37.32 mg/100 g), phlorodzin (2.90–156.22 mg/100 g), ascorbic acid (9.89–54.21 mg/100 g), naringin (1.75–101.82 mg/100 g), *p*‐hydroxy benzoic acid (1.24–2.89 mg/100 g), *o*‐coumaric acid (0.61–3.17 mg/100 g), *p*‐coumaric acid (0.48–3.80 mg/100 g), ferulic acid (1.00–2.23 mg/100 g), epigallocatechin‐3‐gallate (0.97–17.26 mg/100 g), *m*‐coumaric acid (2.01–2.23 mg/100 g), gallic acid (1.69–11.49 mg/100 g) and caffeic acid (0.03–11.46 mg/100 g) (Table 2; Figure S2; Table S3).

**TABLE 2:** Variability in selected phenolic compounds content across the populations of *C. acuminatum* from the Uttarakhand Himalaya.

Population	Catechin		Phlorodzin		Ascorbic acid		Naringin		*p*‐Hydroxy benzoic acid		*o*‐Coumaric acid		*p*‐Coumaric acid		Ferulic	
	AG	BG	AG	BG	AG	BG	AG	BG	AG	BG	AG	BG	AG	BG	AG	BG
**CA1**	18.04 ± 0.07	18.5 ± 0.01	205.37 ± 0.1	30.3 ± 0.06	55.88 ± 0.52	18.13 ± 0.11	7.17 ± 0.03	0 ± 0	1.7 ± 0	0 ± 0	0 ± 0	0 ± 0	3.63 ± 0.27	0.56 ± 0.01	2.4 ± 0	1.33 ± 0.01
**CA2**	20.82 ± 0.97	0 ± 0	195.87 ± 2.85	23.98 ± 6.18	38.64 ± 0.8	43.38 ± 1.74	5.98 ± 0.14	0 ± 0	2.18 ± 0.2	0 ± 0	2.61 ± 0.37	0 ± 0	2.7 ± 0.06	0 ± 0	2.46 ± 0.02	1.11 ± 0
**CA3**	5.57 ± 0.02	29.89 ± 0.2	61.77 ± 8.5	42.14 ± 9.13	6.23 ± 0.08	35.94 ± 0.09	2.49 ± 0.1	2.29 ± 0.01	1.34 ± 0	0 ± 0	0 ± 0	0 ± 0	0 ± 0	1.06 ± 0	0 ± 0	1.91 ± 0
**CA4**	22.41 ± 0.48	19.57 ± 0.05	0 ± 0	12.1 ± 0.27	46.41 ± 0.05	21.58 ± 0.13	7.09 ± 0	0 ± 0	2.37 ± 0.02	1.48 ± 0.1	0 ± 0	0 ± 0	2.21 ± 0.01	0 ± 0	2.11 ± 0.01	1 ± 0.01
**CA5**	5.32 ± 1.88	0 ± 0	84.08 ± 0.14	36.71 ± 0.2	28.38 ± 0.11	48.06 ± 0.18	10.91 ± 0.33	0 ± 0	0 ± 0	2.54 ± 0.1	1.41 ± 0.03	0 ± 0	1.15 ± 0.01	0.97 ± 0	1.45 ± 0.14	1.4 ± 0
**CA6**	0 ± 0	22.42 ± 1.11	403.93 ± 6.59	156.22 ± 14.14	72.18 ± 0.63	44.51 ± 0.42	34.73 ± 1	101.82 ± 68.79	10.54 ± 0.1	1.6 ± 0.02	5.73 ± 0.2	0 ± 0	6.21 ± 0.03	2.13 ± 0.02	9.91 ± 0.17	1.82 ± 0.01
**CA7**	8.12 ± 0.28	28.08 ± 0.2	237.32 ± 2.83	47.26 ± 0.87	49.58 ± 0.17	16.93 ± 0.03	7.51 ± 0.19	0 ± 0	0 ± 0	0 ± 0	3.27 ± 0.01	0.65 ± 0.08	4.67 ± 0.05	0.91 ± 0.01	2.35 ± 0.02	1.61 ± 0.01
**CA8**	0 ± 0	28.43 ± 0.13	413.94 ± 1.83	20.5 ± 0.56	36.87 ± 0.42	21.15 ± 0.07	10.26 ± 0.23	0 ± 0	2.38 ± 0.23	2.1 ± 0.11	0 ± 0	0 ± 0	6.86 ± 0.05	0.49 ± 0	4.89 ± 0.13	1 ± 0.02
**CA9**	2.36 ± 0.02	16.14 ± 0.16	93.89 ± 1.33	28.98 ± 1.7	0 ± 0	22.14 ± 0.3	2.73 ± 0.03	0 ± 0	0 ± 0	0 ± 0	0 ± 0	1.42 ± 0.04	1.92 ± 0	0.88 ± 0.01	1.43 ± 0.02	0 ± 0
**CA10**	0 ± 0	9.7 ± 0.06	451.4 ± 7.8	25.56 ± 1.11	56.01 ± 2.27	23.82 ± 0.83	35.02 ± 0.72	0 ± 0	2.38 ± 0.06	0 ± 0	7.31 ± 0.22	0 ± 0	6.77 ± 0.04	0.48 ± 0	8.77 ± 0.12	1.65 ± 0.06
**CA11**	0 ± 0	25.42 ± 0.75	304.22 ± 0.08	27.58 ± 0.52	0 ± 0	26.49 ± 0.04	9.06 ± 0.05	0 ± 0	1.21 ± 0	1.53 ± 0	5.84 ± 0.08	0 ± 0	0 ± 0	0.5 ± 0	4.95 ± 0.06	1.42 ± 0
**CA12**	1.29 ± 0	0 ± 0	400.44 ± 0.99	0 ± 0	0 ± 0	32.5 ± 8.38	9.25 ± 0.3	0 ± 0	11.28 ± 0.08	1.49 ± 0.01	6.14 ± 1.16	2.19 ± 0.88	8.5 ± 0.09	1.49 ± 0.01	6.8 ± 0.26	1.4 ± 0.02
**CA13**	18.55 ± 0.19	0 ± 0	142.93 ± 3.95	3.54 ± 0.05	23.2 ± 0.08	12.62 ± 1.31	9.95 ± 0.72	0 ± 0	0 ± 0	0 ± 0	1.91 ± 0.06	0 ± 0	2.92 ± 0.03	0 ± 0	3.2 ± 0.01	0 ± 0
**CA14**	29.76 ± 3.29	35.36 ± 0.03	22.16 ± 0.05	49.22 ± 0.21	29.91 ± 0.14	44.74 ± 1.92	0 ± 0	1.91 ± 0.01	0 ± 0	0 ± 0	0 ± 0	0 ± 0	0.57 ± 0.01	0.96 ± 0	1.21 ± 0.01	2.14 ± 0
**CA15**	7.15 ± 0.01	20.53 ± 2.29	43.93 ± 0.05	23.81 ± 0.06	25.63 ± 0.17	13.77 ± 0.16	0 ± 0	0 ± 0	1.57 ± 0	0 ± 0	0 ± 0	0 ± 0	0 ± 0	0 ± 0	5.37 ± 0.03	0 ± 0
**CA16**	0 ± 0	15.9 ± 0.19	325.55 ± 4.91	9.42 ± 0.58	80.23 ± 2.3	21.44 ± 0.29	19.13 ± 1.36	0 ± 0	0 ± 0	0 ± 0	5.97 ± 0.12	0 ± 0	5.18 ± 0.03	0 ± 0	5.74 ± 0.09	0 ± 0
**CA17**	22 ± 0.2	16.68 ± 0.16	35.62 ± 0.45	4.61 ± 0.05	19.08 ± 0.03	30.22 ± 4.46	0 ± 0	0 ± 0	0 ± 0	1.55 ± 0	0 ± 0	0 ± 0	0 ± 0	0 ± 0	0 ± 0	0 ± 0
**CA18**	0 ± 0	12.67 ± 2.3	0 ± 0	0 ± 0	52.91 ± 0.35	21.89 ± 0.14	23.94 ± 0.29	0 ± 0	0 ± 0	2.3 ± 0.4	7.83 ± 0.59	0 ± 0	5.4 ± 0.02	0.53 ± 0	6.7 ± 0.05	1.42 ± 0.01
**CA19**	32.9 ± 0.21	0 ± 0	0 ± 0	0 ± 0	28.52 ± 0.42	54.21 ± 0.45	3.98 ± 0.06	0 ± 0	1.61 ± 0.02	2.89 ± 0.17	1.81 ± 0.04	0 ± 0	2.02 ± 0.02	0 ± 0	2.41 ± 0.03	0 ± 0
**CA20**	6.8 ± 0.07	25.41 ± 0.17	0 ± 0	0 ± 0	32.55 ± 1.73	22.82 ± 0.24	5.36 ± 0.1	2.61 ± 0.05	1.49 ± 0	1.99 ± 0.01	2.45 ± 0.03	0.61 ± 0.04	3.62 ± 0.01	1.12 ± 0	2.21 ± 0.01	1.58 ± 0.02
**CA21**	18.64 ± 3.43	0 ± 0	0 ± 0	0 ± 0	27.26 ± 1.36	41.59 ± 0.17	4.01 ± 0.58	5.47 ± 0.11	1.67 ± 0.04	1.46 ± 0.02	0 ± 0	3.17 ± 0.01	2.3 ± 0.06	3.8 ± 0.08	0 ± 0	2.23 ± 0.03
**CA22**	32.38 ± 3.11	0 ± 0	0 ± 0	0 ± 0	24.41 ± 0.28	44.29 ± 3.97	2.25 ± 0.03	0 ± 0	0 ± 0	1.24 ± 0	0 ± 0	0 ± 0	0.77 ± 0.01	0.86 ± 0	1.8 ± 0.02	1.31 ± 0.01
**CA23**	13.76 ± 0.08	22.36 ± 0.21	72.96 ± 1.42	17.53 ± 0.2	33.84 ± 2.19	27.62 ± 0.62	2.38 ± 0.1	0 ± 0	1.53 ± 0.01	1.63 ± 0	0.67 ± 0.02	0 ± 0	1.59 ± 0.01	0 ± 0	1.33 ± 0.02	0 ± 0
**CA24**	5.19 ± 0.02	37.32 ± 0.36	45 ± 0.8	18.7 ± 0.22	14.9 ± 0.03	46.44 ± 0.15	0 ± 0	0 ± 0	1.5 ± 0.01	0 ± 0	2.06 ± 0.08	0 ± 0	1.22 ± 0.01	0.56 ± 0	0 ± 0	0 ± 0
**CA25**	17.05 ± 0.23	8.83 ± 0.2	156.44 ± 1.86	0 ± 0	51.48 ± 0.47	9.89 ± 0.01	4.43 ± 0.13	3.53 ± 0.03	1.44 ± 0	0 ± 0	0 ± 0	0 ± 0	3.24 ± 0.01	0 ± 0	2.06 ± 0.03	0 ± 0
**CA26**	11.78 ± 0.13	17.93 ± 0.32	53.04 ± 1.07	62.1 ± 1.06	11.76 ± 0.08	18.47 ± 0.19	0 ± 0	0 ± 0	1.32 ± 0.02	0 ± 0	0.8 ± 0.06	0 ± 0	1.73 ± 0.02	1.49 ± 0.02	0 ± 0	0 ± 0
**CA27**	0 ± 0	0 ± 0	123.36 ± 1.51	21.06 ± 0.33	33.08 ± 0.29	18.2 ± 1.19	1.74 ± 0.09	0 ± 0	1.51 ± 0	0 ± 0	2.71 ± 0.05	0 ± 0	1.82 ± 0.01	0 ± 0	0.01 ± 0	0 ± 0
**CA28**	10.91 ± 1.15	0 ± 0	57.18 ± 0.99	0 ± 0	17.09 ± 0.06	0 ± 0	1.82 ± 0.11	0 ± 0	1.34 ± 0.01	0 ± 0	0.71 ± 0.02	0 ± 0	0.99 ± 0.01	0.75 ± 0.02	0 ± 0	0 ± 0
**CA29**	0 ± 0	16.75 ± 0.08	0 ± 0	0 ± 0	53.23 ± 0.2	18.6 ± 0.17	6.47 ± 0.22	1.75 ± 0.12	0 ± 0	1.89 ± 0.01	7.59 ± 0.24	1.88 ± 0.13	4.13 ± 0.03	1.26 ± 0.01	4.77 ± 0.05	1.1 ± 0.02
**CA30**	11.33 ± 0.27	13.31 ± 0.1	3.35 ± 0.01	2.9 ± 0.03	29.39 ± 0.86	26.73 ± 0.14	9.29 ± 0.16	4.39 ± 0.05	2.01 ± 0.01	1.42 ± 0	0 ± 0	0 ± 0	0 ± 0	0 ± 0	0 ± 0	0 ± 0
**CA31**	2.95 ± 0.04	0 ± 0	3.41 ± 0.08	3.04 ± 0.06	17.55 ± 0.1	22.13 ± 0.5	3.99 ± 0.02	4.46 ± 0.04	1.32 ± 0	0 ± 0	0 ± 0	0 ± 0	0 ± 0	0 ± 0	0 ± 0	0 ± 0
**CA32**	22.78 ± 0.23	0 ± 0	16.06 ± 0.31	22.2 ± 0.76	49.72 ± 0.74	47.53 ± 1.06	23.19 ± 0.39	43.12 ± 0.84	1.57 ± 0.04	0 ± 0	0 ± 0	0 ± 0	0 ± 0	0 ± 0	0 ± 0	0 ± 0
**CA33**	0 ± 0	0 ± 0	11.89 ± 0.33	3.68 ± 0.02	33.41 ± 0.14	40.21 ± 0.44	0 ± 0	6.44 ± 0.17	1.34 ± 0	0 ± 0	0 ± 0	0 ± 0	0 ± 0	0 ± 0	0 ± 0	0 ± 0
**CA34**	44.51 ± 1	9.39 ± 1.23	0 ± 0	0 ± 0	42.01 ± 0.24	18.16 ± 0.33	0 ± 0	34.09 ± 1.21	0 ± 0	1.37 ± 0.01	0 ± 0	0 ± 0	0 ± 0	0 ± 0	0 ± 0	0 ± 0
**CA35**	0 ± 0	14.3 ± 0.19	15.49 ± 5.24	0 ± 0	66.81 ± 1.32	18.92 ± 0.16	74.06 ± 16.93	0 ± 0	19.02 ± 6.91	1.36 ± 0.02	0 ± 0	0 ± 0	0 ± 0	1.85 ± 0.01	0 ± 0	0 ± 0

*Note*: Given values in g/100 g; values are means ± standard error (SE). Within columns, SE values followed by different superscript letters (a, b, c, etc.) indicate significant differences (p<0.05) according to One‐way ANOVA followed by Duncan multiple range test (DMRT).

Abbreviations: AG, above ground part; BG, below ground part.

### 3.6 Identification of Candidate Elite Populations on the Basis of Studied Parameters

Among all the thirty‐five studied populations of the species belonging to different habitat and altitudinal classes, Thalkedar population exhibited comparatively higher values for several morphological traits, including plant height (30.93 ± 1.12 cm), stem length (7.12 ± 0.58 cm), stem width (1.64 ± 0.03 cm), pseudobulb length (6.12 ± 0.21 cm), pseudobulb width (2.29 ± 0.07 cm), leaf length (15.02 ± 0.24 cm) and leaf width (5.45 ± 0.06 cm). Whereas the Lohaghat population was reported to have higher reproductive performance with a higher inflorescence length (16.64 ± 0.85 cm) as well as a higher number of flowers (16.2 ± 1.41). Similarly, for nutritional, phytochemical, and antioxidant properties, the Pandukholi population exhibited comparatively higher carbohydrate content in the root part (45.65 ± 1.52%), flavonoid content in the aerial parts (2.09 ± 0.26 mg QE/g), and also higher DPPH antioxidant activity in the root part (1.33 ± 0.09 mM AAE/100 g DW). Also, the Thalkedar population showed relatively higher content of protein in aerial parts (4.28 ± 0.32%), phenolics in root part (2.13 ± 0.15 mg GAE/g), as well as the FRAP antioxidant activity in the aerial part (6.51 ± 0.30 mM AAE/100 g DW). In terms of phenolic compounds, the populations with the highest contents, such as Mukteshwar (highest catechin), Kalika (exceptionally high phlorodzin), Mallroad (ascorbic acid and E‐GCG), Majkhali (naringin and ferulic acid), Raditop (naringin and *p*‐hydroxy benzoic acid) and Chakrata (caffeic acid and m‐coumaric acid), can be designated as candidate elite populations for further validation as chemotypes and can further be multiplied for use in pharmaceuticals.

## 4 Discussion

Plant species adapt their morphological and physiological characteristics in response to environmental variations [20]. These variations can be adaptive, defensive, or submissive, or lead to diverse responses based on species. In this context, the Thalkedar (2040 m, Oak‐dominated habitat's vegetation) population demonstrated relatively higher plant height, stem length, stem width, leaf length, leaf width, pseudobulb length, and width. These results suggest that the Thalkedar population has better adaptation to local conditions. The propagules of these populations could be used for mass cultivation to fulfil the demand of the pharmaceutical industry and for conservation of the species [21]. Also, the sites supporting better morphological growth of the plants should be protected for conservation, as they possess the elite germplasm of the species and have the required microclimatic habitats for the species, which is also supported by previous studies [22, 23, 24]. Studies indicated that variations in quantitative characters among populations are mostly influenced by various environmental factors, controlled by various genes, and are easily influenced by these environmental alterations [25]. Differences in population traits across sites are mostly due to genetic differentiation or microclimatic variations, often pointing to differences in genes [16, 26]. Similar morphological variations in wild populations have also been reported for several species in the Himalayan region, like *Justicia adhatoda* [27], *Colchicum luteum* [28], *Viola canescens* [25], *Rosa* L. [29], *Rheum australe* [30], *Polygonatum verticillatum* [31], *Trillium govanianum* [32], and *Viola* species [33]. The study found no correlation between altitude and the morphological characteristics of the species. Elevation gradients create natural climatic variation, affecting key environmental factors like atmospheric temperature, CO_2_ partial pressure, solar irradiance, and rainfall within confined areas [34].

Plants are a source of important bioactive constituents like phenolic acids, flavonoids, and vitamins providing health benefits, due to which these plants are used in various medicines, food, and also in the pharmaceutical industry as the raw material [35, 36, 37]. Thus, a detailed investigation of the nutritional and phytochemical components of the species showed that the carbohydrate content ranged from 8.6% to 45.65% in the studied populations of the species. The present findings align with previous research studies on herbal plants (*Menthae folia*, *Taraxacum radices*, *Urticae folia*, and *Rosae frucrus* [38]). Similarly, the protein content ranged between 1.27% and 4.28%, which is in agreement with the studies on other medicinal plant species [*Centella asiatica* (3.1%), *Ocimum tenuiflorum* (2.3%) [39]; *Ocimum gratissimum* (5.2%), *Ipomoea aquatica* (3.5%), and *Bupleurum smithii* (1.98%) [40]]. While the methionine content ranged between 3.31% and 9.86%, a maximum (9.86%) was recorded in the Ramgarh (1980 m, oak‐dominated habitat vegetation) population (root parts) and a minimum (3.31%) in the Sirakot (2042 m, mixed broadleaf forest) population (root parts). The present findings revealed a higher methionine content than previously reported in other medicinal plants [41]. Studies showed that the higher the abiotic stress on the plant, the higher the amino acid content will be present [42]. Here, the high values for methionine thus suggest that the studied populations are under high stress in these wild habitats.

For the phytochemical content, total phenolics (0.28–2.13 mg GAE/g DW), flavonoids (0.17–2.09 mg QE/g DW) and total tannin (0.38–6.95 mg TAE/g DW) content were recorded. A similar range for the concentration of various phytochemicals has also been previously reported in other such species, like *Valeriana jatamansi* [18], *Trillium govanianum* [32], *Polygonatum verticillatum* [31], *Rheum australe* [30], *Ocimum gratissimum* and *Solanum aethiopicum* [43]. It shows that the oak‐dominated natural habitats and a higher elevation range reported a higher phytochemical diversity than other habitat conditions at lower elevations. Plant species growing under different environmental conditions alter their structural and functional properties for survival and defense [44]. This, in turn, affects the production of various compounds, including phenolics and flavonoids, and so forth [45, 46], which justifies the variations in phytochemical content in the studied populations of the target species.

Elevation could be the key predictor of plant phytochemical defense strategies, influencing their biochemical properties and composition. Altitude, in relation to various microclimatic environmental conditions and genetic variations, influences the biochemical and biological properties of plants and their phytochemical composition [15, 26].

## 5 Conclusion

*C. acuminatum*, a therapeutic herb, was studied in this detailed investigation to determine how its morphology, secondary metabolite composition, nutritional content, and antioxidant activities vary with respect to elevation and habitat change. Thirty‐five diverse populations of the species from Uttarakhand, Western Himalaya, were analyzed. The findings of the study suggested that various environmental factors significantly influence the quality and concentrations of various bioactive compounds present in *C. acuminatum*, which is important for its use in the pharmaceutical industry and modern medicine. Therefore, mass multiplication using nodal segments and various *in vitro* culture techniques is recommended for the species to fulfil the demand and continuous supply of bioactive compounds, thus supporting trait improvement, breeding programmes, sustainable utilization, and conservation of this valuable bioresource.

## Author Contributions

**Kalpana Rautela**: investigation, formal analysis, data curation, Writing – original draft. **Arun K. Jugran**: conceptualization, supervision, resources, funding acquisition, writing – review & editing. **Amit Bahukhandi**: formal analysis, methodology, software. **I.D. Bhatt**: supervision, resources, writing – review & editing. **Prem Prakash**: supervision, writing – review & editing.

## Funding

This work was supported by the National Medicinal Plant Board (NMPB), Department of AYUSH, New Delhi (F. No. Z.18017/187/CSS/R&D/UK‐01/2019‐20‐NMPB‐IV A), and G.B. Pant National Institute of Himalayan Environment and Sustainable Development (GBP‐NIHE) under In‐house project No. 04 (Mainstreaming Himalayan Biodiversity for Sustainable Development).

## Conflicts of Interest

The authors declare no conflicts of interest.

## Use of Generative AI and AI‐Assisted Technologies in the Writing Process

The authors declare no use of any Artificial Intelligence Generated Content (AIGC) tools such as ChatGPT and others based on large language models (LLMs) in developing any portion of this manuscript.

## Supporting information




**Supporting File 1**: cbdv71662‐sup‐0001‐SuppMat.docx.

## Data Availability

All data supporting the findings of this study are available in the form of tables and figures, and Supporting Information with the manuscript.
