# Supplementary material for: Altitudinal and Habitat‐Dependent Morphological and Biochemical Variations in Crepidium acuminatum (D.Don) Szlach. in the Western Himalaya
Source: Chem Biodivers. 2026 Sep 21;23(9):e71662. doi: 10.1002/cbdv.71662 (PMC13592693; doi:10.1002/cbdv.71662)
Supplement: Supplementary file 1 [file CBDV-23-e71662-s001.docx]

**List of supplementary data**

**Figure S1** Study sites for data collection of *C. acuminatum* in Uttarakhand, Western Himalaya

**Figure S2** HPLC chromatogram: (A) HPLC chromatogram of standard reference compounds, (B - E) HPLC chromatogram of samples: 1. CA1 (AG), 2. CA1 (BG), 3. CA2 (AG), 4. CA2 (BG), 5. CA3 (AG), 6. CA3 (BG), 7. CA4 (AG), 8. CA4 (BG), 9. CA5 (AG), 10. CA5 (BG), 11. CA6 (AG), 12. CA6 (BG), 13. CA7 (AG), 14. CA7 (BG), 15. CA8 (AG), 16. CA8 (BG), 17. CA9 (AG), 18. CA9 (BG), 19. CA10 (AG),20. CA10 (BG), 21. CA11 (AG), 22. CA11 (BG), 23. CA12 (AG), 24. CA12 (BG), 25. CA13 (AG), 26. CA13 (BG), 27. CA14 (AG), 28. CA14 (BG), 29. CA15 (AG), 30. CA15 (BG), 31. CA16 (AG), 32. CA16 (BG), 33. CA17 (AG), 34. CA17 (BG), 35. CA18 (AG), 36. CA18 (BG), 37. CA19 (AG), 38. CA19 (BG), 39. CA20 (AG), 40. CA20 (BG), 41. CA21 (AG), 42. CA21 (BG), 43. CA22 (AG), 44. CA22 (BG), 45. CA23 (AG), 46. CA23 (BG), 47. CA24 (AG), 48. CA24 (BG), 49. CA25 (AG), 50. CA25 (BG), 51. CA26 (AG), 52. CA26 (BG), 53. CA27 (AG), 54. CA27 (BG), 55. CA28 (AG), 56. CA28 (BG), 57. CA29 (AG), 58. CA29 (BG), 59. CA30 (AG), 60. CA30 (BG), 61. CA31 (AG), 62. CA31 (BG), 63. CA32 (AG), 64. CA32 (BG), 65. CA33 (AG), 66. CA33 (BG), 67. CA34 (AG), 68. 34 (BG), 69. CA35 (AG), 70. CA35 (BG) (*Note: AG = Above ground sample, BG = Below ground sample, and CA1, CA2 & so on indicate the populations as given in Table S1)

(**Note:** The HPLC validation methodology was developed and standardised by following Singh et al. 2026 [Singh, B., Singh, L., Kewlani, P., & Bhatt, I. D. (2026). Harnessing the potential of selected Himalayan species for phyto-cosmeceutical formulations guided through biochemometric analysis. *Scientific Reports*, *16*(1), 5928]).

**Table S1** Study area of the study in the Uttarakhand Himalaya

| **S. No.** | **Code** | **Study Site** | **Latitude (°N)** | **Longitude (°E)** | **Elevation (m asl)** | **Habitat** |
| --- | --- | --- | --- | --- | --- | --- |
| 1 | CA1 | Lweshal | 29°29'06" | 79°40'51" | 1526 | Oak-Pine mixed forest |
| 2 | CA2 | Lohaghat | 29°23'58" | 80°04'45" | 1624 | Deodar forest |
| 3 | CA3 | Bhowali | 29°23'19" | 79°30'31" | 1650 | Oak-pine mixed forest |
| 4 | CA4 | Mayawati | 29°23'17" | 80°03'55" | 1696 | Banj Oak-Rhododendron mixed forest |
| 5 | CA5 | Lamgarha | 29°34'94" | 79°41'21" | 1713 | Banj Oak-Rhododendron mixed forest |
| 6 | CA6 | Majkhali | 29°40'42'' | 79°31'01'' | 1714 | Banj-Deodar-Pine |
| 7 | CA7 | Kausani | 29°50'35" | 79°36'04" | 1750 | Banj-Deodar-Pine |
| 8 | CA8 | Gangolihat | 29°39'41" | 80°2'21.4" | 1765 | Mixed forest |
| 9 | CA9 | Salyura | 29°16'19" | 79°39'05" | 1774 | Banj Oak mixed forest |
| 10 | CA10 | Kalika | 29°39'54'' | 79°28'40'' | 1778 | Banj Oak-Pine |
| 11 | CA11 | Dol Ashram | 29°30'20" | 79°45'28" | 1789 | Banj Oak-Rhododendron mixed forest |
| 12 | CA12 | Nagdev forest | 30°8'7.3" | 78°46'39.0" | 1870 | Banj Oak-Deodar mixed forest |
| 13 | CA13 | Jakholi | 30°23'43'' | 78°53'35'' | 1875 | Oak mixed forest |
| 14 | CA14 | Chandak | 29°36'11.3" | 80°11'24.2" | 1880 | Banj Oak-Deodar mixed forest |
| 15 | CA15 | Khirshu | 30°10'16.1" | 78°52'28.4" | 1882 | Banj Oak-Deodar mixed forest |
| 16 | CA16 | Mallroad | 29°37'14'' | 79°26'31'' | 1893 | Chir Pine-Banj oak |
| 17 | CA17 | Jarmola | 30°57'04'' | 78°06'36'' | 1901 | Mixed-Oak Forest |
| 18 | CA18 | Hanumangarhi | 29°36'57'' | 79°45'33" | 1919 | Mixed-Oak Forest |
| 19 | CA19 | Devidhura | 29°24’46’’ | 79°51’55’’ | 1922 | Banj Oak-Pine |
| 20 | CA20 | Jageshwar | 29°38'11" | 79°50'55" | 1940 | Deodar forest |
| 21 | CA21 | Madhyamaheshwar | 30°37'43'' | 79°12'43'' | 1960 | Banj Oak-Pine |
| 22 | CA22 | Chaubattia | 29°37'14'' | 79°27'44'' | 1961 | Deodar-Banj Oak |
| 23 | CA23 | Sandev forest | 29°48'54.3" | 80°14'32.9" | 1976 | Banj Oak Forest |
| 24 | CA24 | Ramgarh | 29°25'16'' | 79°33'14'' | 1980 | Oak forest |
| 25 | CA25 | Farsundakhal | 30°10'5.0" | 78°52'32.2" | 2033 | Banj Oak Forest |
| 26 | CA26 | Thalkedar | 29°32'01" | 80°14'32" | 2040 | Banj Oak Forest |
| 27 | CA27 | Sirakot | 29°48'57.9" | 80°14'36.5" | 2042 | Banj Oak-Rhododendron mixed forest |
| 28 | CA28 | Berinag | 29°50'37" | 80°02'07" | 2050 | Banj Oak Forest |
| 29 | CA29 | Pandukholi | 29°50'22'' | 79°27'61'' | 2100 | Banj Oak |
| 30 | CA30 | Syahidevi | 29°34'43" | 79°33'31" | 2113 | Mixed forest |
| 31 | CA31 | Doonagiri | 29°48'31'' | 79°27'32'' | 2157 | Deodar-Banj Oak |
| 32 | CA32 | Chakrata | 30°41'50" | 77°52'14" | 2151 | Banj Oak-Rhododendron mixed forests |
| 33 | CA33 | Munsiyari | 30°05'28" | 80°14'19" | 2200 | Banj Oak Forest |
| 34 | CA34 | Mukteshwar | 29°26'12'' | 79°38'58'' | 2249 | Oak mixed forest |
| 35 | CA35 | Raditop | 30°46'16" | 78°15'27" | 2280 | Mixed Oak Forest |

**Table S2** Variability in nutritional, phytochemical and antioxidant activity across the populations of *C. acuminatum* from the Uttarakhand Himalaya

| **Population** | **Carbohydrate** | | **Protein** | | **Methionine** | | **Phenol** | | **Flavonoid** | | **Tannin** | | **DPPH** | | **FRAP** | |
| --- | --- | --- | --- | --- | --- | --- | --- | --- | --- | --- | --- | --- | --- | --- | --- | --- |
|  | **AG** | **BG** | **AG** | **BG** | **AG** | **BG** | **AG** | **BG** | **AG** | **BG** | **AG** | **BG** | **AG** | **BG** | **AG** | **BG** |
| **CA1** | 34.26 ± 0.61^b^ | 14.1 ± 0.67^o^ | 1.57 ± 0.14^n^ | 1.37 ± 0.08^m^ | 7.44 ± 0.42^abcdef^ | 4.77 ± 0.28^ghijkl^ | 0.32 ± 0.04^l^ | 0.45 ± 0.05^lm^ | 0.72 ± 0.07^jk^ | 0.17 ± 0.03^k^ | 1.24 ± 0.09^jkl^ | 0.72 ± 0.05^opqrst^ | 0.29 ± 0.02^hij^ | 0.24 ± 0.03^hijk^ | 1.05 ± 0.03^no^ | 1.06 ± 0.05^jklmn^ |
| **CA2** | 15.44 ± 0.68^lmno^ | 16.52 ± 0.59^no^ | 1.62 ± 0.35^n^ | 1.27 ± 0.04^m^ | 7.86 ± 0.13^abc^ | 5.18 ± 0.29^cdefghij^ | 0.28 ± 0.02^l^ | 0.37 ± 0.02^m^ | 0.64 ± 0.15^jk^ | 0.33 ± 0.03^jk^ | 1.13 ± 0.07^jklm^ | 0.88 ± 0.11^lmnopqrs^ | 0.13 ± 0.02^ij^ | 0.11 ± 0.01^jk^ | 1.01 ± 0.01^o^ | 0.84 ± 0.2^mn^ |
| **CA3** | 13.77 ± 0.79^nop^ | 21.06 ± 2.3^jklmn^ | 1.88 ± 0.21^klmn^ | 1.68 ± 0.41^ijkl^ | 7.75 ± 0.24^abcd^ | 4.72 ± 0.43^ghijkl^ | 0.48 ± 0.05^hijk^ | 0.36 ± 0.03^m^ | 0.65 ± 0.08^jk^ | 0.36 ± 0.02^ijk^ | 0.71 ± 0.05^mn^ | 1.05 ± 0.03^klmnop^ | 0.22 ± 0.03^ij^ | 0.16 ± 0.01^ijk^ | 1.26 ± 0.02^lmno^ | 0.95 ± 0.05^lmn^ |
| **CA4** | 13.68 ± 0.45^nop^ | 26.71 ± 1.99^ghij^ | 1.72 ± 0.24^mn^ | 1.75 ± 0.27^hijkl^ | 6.31 ± 0.85^cdefghi^ | 5.87 ± 0.12^bcdefgh^ | 0.34 ± 0.05^jk^ | 0.5 ± 0^lm^ | 0.65 ± 0.07^jk^ | 0.59 ± 0.03^cdefghij^ | 1.21 ± 0.18^jklm^ | 1.91 ± 0.1^fg^ | 0.1 ± 0.003^j^ | 0.24 ± 0.03^hijk^ | 1.1 ± 0.1^no^ | 0.71 ± 0.05^n^ |
| **CA5** | 15.75 ± 0.32^lmno^ | 18.07 ± 2.96^mno^ | 1.77 ± 0.06^lmn^ | 1.6 ± 0.25^lm^ | 5.64 ± 0.17^fghi^ | 3.76 ± 0.4^kl^ | 0.35 ± 0.08^jk^ | 0.45 ± 0.04^lm^ | 0.48 ± 0.19^k^ | 0.66 ± 0.02^cdefghi^ | 0.72 ± 0.03^mn^ | 0.54 ± 0.06^st^ | 0.34 ± 0.03^ghij^ | 0.23 ± 0.03^ijk^ | 1.17 ± 0.09^mno^ | 1.03 ± 0.02^klmn^ |
| **CA6** | 15.73 ± 0.41^lmno^ | 32.31 ± 1.38^defg^ | 1.81 ± 0.11^klmn^ | 1.64 ± 0.11^klm^ | 6.47 ± 0.23^bcdefgh^ | 4.49 ± 0.26^hijkl^ | 0.44 ± 0.03^ijk^ | 0.56 ± 0.01^lm^ | 0.96 ± 0.1^ijk^ | 0.4 ± 0.06^hijk^ | 1.19 ± 0.04^jklm^ | 1.27 ± 0.06^ijkl^ | 0.45 ± 0.09^fghi^ | 0.35 ± 0.03^fghijk^ | 0.96 ± 0.16^o^ | 1.09 ± 0.12^jklmn^ |
| **CA7** | 20.5 ± 1.09^fg^ | 19.22 ± 3.39^lmno^ | 1.83 ± 0.33^klmn^ | 2.44 ± 0.11^efgh^ | 7.15 ± 0.3^bcdefg^ | 4.93 ± 0.44^fghijkl^ | 0.37 ± 0.01^jk^ | 0.84 ± 0.05^hijklm^ | 0.8 ± 0.05^jk^ | 0.44 ± 0.08^ghijk^ | 1.45 ± 0.03^ijk^ | 2.19 ± 0.11^ef^ | 0.4 ± 0.05^fghij^ | 0.02 ± 0.01^k^ | 1.42 ± 0.02^jklmno^ | 1.31 ± 0.09^hijklm^ |
| **CA8** | 17.26 ± 1.83^hijkl^ | 19.51 ± 0.54^lmno^ | 1.87 ± 0.07^klmn^ | 1.69 ± 0.06^ijkl^ | 6.81 ± 0.22^bcdefgh^ | 5.04 ± 0.35^efghijk^ | 0.5 ± 0^hijk^ | 0.78 ± 0.1^jklm^ | 0.94 ± 0.05^ijk^ | 0.48 ± 0.05^efghij^ | 4 ± 0.31^c^ | 2.26 ± 0.02^e^ | 0.4 ± 0.06^fghij^ | 0.3 ± 0.05^ghijk^ | 1.3 ± 0.05^klmno^ | 1.22 ± 0.14^ijklm^ |
| **CA9** | 19.68 ± 0.69^fghi^ | 20.63 ± 0.96^klmn^ | 1.74 ± 0.04^mn^ | 1.73 ± 0.32^hijkl^ | 7.61 ± 0.23^abcde^ | 5.54 ± 0.23^cdefghij^ | 0.57 ± 0.09^hijk^ | 0.63 ± 0.23^klm^ | 0.91 ± 0.05^ijk^ | 0.33 ± 0.01^jk^ | 1.19 ± 0.14^jklm^ | 0.74 ± 0.01^nopqrst^ | 0.41 ± 0.06^fghi^ | 0.32 ± 0.04^ghijk^ | 1.4 ± 0.1^jklmno^ | 1.09 ± 0.06^jklmn^ |
| **CA10** | 8.6 ± 0.82^r^ | 19.96 ± 0.89^lmno^ | 1.95 ± 0.1^jklmn^ | 1.68 ± 0.17^ijkl^ | 5.95 ± 1.23^defghi^ | 8.86 ± 0.61^a^ | 0.55 ± 0.02^hijk^ | 0.56 ± 0.09^lm^ | 1.02 ± 0.01^ghij^ | 0.45 ± 0.05^fghijk^ | 2.35 ± 0.07^def^ | 0.91 ± 0.03^lmnopqrs^ | 0.43 ± 0.03^fghi^ | 0.52 ± 0.04^cdefghi^ | 1.42 ± 0.1^jklmno^ | 1.37 ± 0.08^hijkl^ |
| **CA11** | 17.75 ± 0.22^hijkl^ | 22.13 ± 1.76^jklmn^ | 2.03 ± 0.29^ijklmn^ | 3.62 ± 0.04^a^ | 6.63 ± 0.62^bcdefgh^ | 6.44 ± 0.65^bcdef^ | 0.53 ± 0.02^hijk^ | 0.75 ± 0.17^jklm^ | 0.96 ± 0.14^ijk^ | 0.66 ± 0.02^cdefghi^ | 1.59 ± 0.14^hij^ | 1.44 ± 0.05^hij^ | 0.57 ± 0.07^efgh^ | 0.48 ± 0.02^defghi^ | 1.37 ± 0.15^jklmno^ | 1.37 ± 0.12^hijkl^ |
| **CA12** | 13.7 ± 1.09^nop^ | 17.1 ± 1.41^no^ | 2.27 ± 0.1^ghijklmn^ | 1.83 ± 0.03^hijkl^ | 7.22 ± 0.12^bcdefg^ | 4.15 ± 0.33^ijkl^ | 0.57 ± 0.03^hijk^ | 0.87 ± 0.06^ghijklm^ | 0.72 ± 0.12^jk^ | 0.44 ± 0.06^ghijk^ | 1.72 ± 0.09^ghi^ | 0.56 ± 0.06^rst^ | 0.44 ± 0.09^fghi^ | 0.44 ± 0.09^efghij^ | 1.44 ± 0.27^jklmno^ | 1.48 ± 0.12^ghijk^ |
| **CA13** | 40.95 ± 0.35^a^ | 23.21 ± 2.06^ijklm^ | 2.08 ± 0.34^hijklmn^ | 1.84 ± 0.07^hijkl^ | 6.26 ± 0.34^cdefghi^ | 4.4 ± 0.14^hijkl^ | 0.68 ± 0.04^ghijk^ | 0.8 ± 0.01^ijklm^ | 1.34 ± 0.18^efghi^ | 0.57 ± 0.08^cdefghij^ | 2.51 ± 0.21^de^ | 0.59 ± 0.06^qrst^ | 0.36 ± 0.03^fghij^ | 0.41 ± 0.05^efghij^ | 1.66 ± 0.23^ijklmn^ | 1.24 ± 0.03^ijklm^ |
| **CA14** | 17.66 ± 2.39^hijkl^ | 19.34 ± 0.73^lmno^ | 3.5 ± 0.1^bc^ | 1.6 ± 0.16^lm^ | 6.75 ± 0.87^bcdefgh^ | 4.36 ± 0.13^hijkl^ | 1.22 ± 0.1^cdef^ | 1.14 ± 0.01^fghijk^ | 0.97 ± 0.01^ijk^ | 1.21 ± 0.01^a^ | 1.33 ± 0.09^ijk^ | 1.87 ± 0.09^fg^ | 0.4 ± 0.01^fghij^ | 0.23 ± 0.01^ijk^ | 2.09 ± 0.13^ghi^ | 1.41 ± 0.3^hijkl^ |
| **CA15** | 21.42 ± 0.17^ef^ | 25.12 ± 1.04^hijkl^ | 2.91 ± 0.14^cdefg^ | 1.88 ± 0.07^ghijkl^ | 5.13 ± 0.52^hi^ | 6.73 ± 0.69^bc^ | 0.96 ± 0.03^efgh^ | 0.94 ± 0.12^ghijkl^ | 1 ± 0.1^hij^ | 0.59 ± 0.06^cdefghij^ | 1.17 ± 0.05^jklm^ | 1.04 ± 0.004^klmnop^ | 0.42 ± 0.08^fghi^ | 0.59 ± 0.06^bcdefgh^ | 1.8 ± 0.15^ijklm^ | 1.47 ± 0.18^ghijkl^ |
| **CA16** | 14.49 ± 0.07^mnop^ | 19.72 ± 0.25^lmno^ | 2.38 ± 0.19^ghijklm^ | 2.44 ± 0.09^efgh^ | 9.02 ± 0.89^a^ | 4.46 ± 0.48^hijkl^ | 0.7 ± 0.06^ghijk^ | 0.65 ± 0.1^klm^ | 0.92 ± 0.05^ijk^ | 0.38 ± 0.03^ijk^ | 1.02 ± 0.1^klmn^ | 0.38 ± 0.05^t^ | 0.59 ± 0.02^efgh^ | 0.61 ± 0.2^bcdefg^ | 2.41 ± 0.0^fgh^ | 1.63 ± 0.19^ghi^ |
| **CA17** | 19.8 ± 0.79^fgh^ | 17.31 ± 1.95^mno^ | 2.4 ± 0.26^ghijklm^ | 2.11 ± 0.31^fghijk^ | 5.8 ± 0.54^efghi^ | 3.92 ± 0.33^jkl^ | 0.83 ± 0.05^fghij^ | 0.63 ± 0.13^klm^ | 1.33 ± 0.37^efghi^ | 0.68 ± 0.1^cdefghi^ | 2.71 ± 0.17^d^ | 0.81 ± 0.06^mnopqrs^ | 0.62 ± 0.09^defg^ | 0.71 ± 0.22^bcde^ | 1.76 ± 0.13^ijklm^ | 1.49 ± 0.07^ghijk^ |
| **CA18** | 16.01 ± 1.46^klmn^ | 34.94 ± 0.86^bcde^ | 2.49 ± 0.27^fghijkl^ | 2.77 ± 0.19^cdef^ | 6.3 ± 0.3 ^cdefghi^ | 4.93 ± 0.32^fghijkl^ | 0.9 ± 0.17^fghi^ | 1.23 ± 0.07^efghij^ | 1.11 ± 0.12^fghij^ | 0.61 ± 0.07^cdefghij^ | 2.14 ± 0.28^efg^ | 1.14 ± 0.01^jklmn^ | 0.77 ± 0.12^cde^ | 0.68 ± 0.2^bcdef^ | 1.76 ± 0.21^ijklm^ | 1.39 ± 0.11^hijkl^ |
| **CA19** | 25.52 ± 0.58^d^ | 26.69 ± 0.67^ghij^ | 2.55 ± 0.06^fghijk^ | 2.34 ± 0.45^efghij^ | 7.25 ± 1.22^abcdefg^ | 6.65 ± 0.98^bcd^ | 0.91 ± 0.16^fghi^ | 1.14 ± 0.24_fghijk_ | 1.13 ± 0.05^efghij^ | 0.64 ± 0.09^cdefghij^ | 1.77 ± 0.17^ghi^ | 4.21 ± 0.23^b^ | 0.67 ± 0.03^def^ | 0.68 ± 0.17^bcdef^ | 1.54 ± 0.32^ijklmno^ | 2.85 ± 0.07^b^ |
| **CA20** | 21.68 ± 0.13^ef^ | 28.22 ± 3.52^fghi^ | 2.61 ± 0.22^efghij^ | 2.38 ± 0.09^efghi^ | 7.15 ± 0.33^bcdefg^ | 6.2 ± 1.03^bcdefg^ | 0.95 ± 0.18^efgh^ | 1.23 ± 0.15^efghij^ | 1.08 ± 0.1^fghij^ | 0.34 ± 0.1^jk^ | 5.52 ± 0.2^a^ | 1.57 ± 0.09^ghi^ | 0.75 ± 0.09^cde^ | 0.7 ± 0.05^bcdef^ | 1.84 ± 0.17^hijkl^ | 1.73 ± 0.15^fghi^ |
| **CA21** | 18.42 ± 0.48^ghijk^ | 25.77 ± 0.8^hijk^ | 2.53 ± 0.24^fghijk^ | 2.39 ± 0.08^efghi^ | 5.75 ± 0.66^efghi^ | 4.73 ± 0.29^ghijkl^ | 0.67 ± 0.1^ghijk^ | 1.42 ± 0.16^cdefg^ | 1.31 ± 0.06^efghi^ | 0.53 ± 0.09^defghij^ | 1.96 ± 0.07^fgh^ | 0.97 ± 0.07^lmnopq^ | 0.76 ± 0.06^cde^ | 0.7 ± 0.05^bcdef^ | 1.9 ± 0.15^hijk^ | 1.8 ± 0.16^efgh^ |
| **CA22** | 13.2 ± 0.2^op^ | 26.23 ± 3.2^hijk^ | 2.71 ± 0.46^defghi^ | 1.52 ± 0.1^lm^ | 7.31 ± 0.53^abcdef^ | 4.96 ± 0.54^fghijk^ | 0.96 ± 0.07^efgh^ | 1.34 ± 0.5^cdefghi^ | 0.98 ± 0.06^ijk^ | 0.67 ± 0.09^cdefghi^ | 0.95 ± 0.05^klmn^ | 1.1 ± 0.22^jklmno^ | 0.79 ± 0.06^cde^ | 0.61 ± 0.06^bcdefg^ | 3.3 ± 0.55^cd^ | 1.83 ± 0.35^efgh^ |
| **CA23** | 21.32 ± 0.57^ef^ | 28.46 ± 1.21^fghi^ | 2.8 ± 0.29^cdefgh^ | 2.22 ± 0.15^efghijk^ | 5.43 ± 0.13^ghi^ | 3.56 ± 0.66^kl^ | 1.08 ± 0.13^defg^ | 1.29 ± 0.04^defghij^ | 1.56 ± 0.05^cdef^ | 0.63 ± 0.04^cdefghij^ | 2.09 ± 0.17^efg^ | 1.01 ± 0.03^lmnop^ | 0.79 ± 0.05^cde^ | 0.72 ± 0.1^bcde^ | 1.99 ± 0.16^hij^ | 2.32 ± 0.06^cd^ |
| **CA24** | 18.75 ± 0.29^ghij^ | 30.15 ± 2.33^efgh^ | 3.17 ± 0.12^bcdef^ | 2.56 ± 0.16^defg^ | 4.63 ± 0.44^i^ | 9.86 ± 0.25^a^ | 1.12 ± 0.21^defg^ | 1.39 ± 0.03^cdefgh^ | 1.31 ± 0.35^efghi^ | 0.77 ± 0.01^cde^ | 0.9 ± 0.04^lmn^ | 1.16 ± 0.04^jklm^ | 0.64 ± 0.09^defg^ | 0.73 ± 0.07^bcde^ | 1.66 ± 0.13^ijklmn^ | 2.13 ± 0.09^cdef^ |
| **CA25** | 34.68 ± 0.26^b^ | 28.44 ± 0.38^fghi^ | 3.28 ± 0.24^bcde^ | 2.7 ± 0.23^cdef^ | 5.77 ± 0.39^efghi^ | 6.58 ± 0.38^bcde^ | 1.15 ± 0.39^defg^ | 1.23 ± 0.42^efghij^ | 1.42 ± 0.12^defghi^ | 0.72 ± 0.04^cdefg^ | 0.86 ± 0.01^lmn^ | 0.78 ± 0.06^mnopqrs^ | 1.29 ± 0.01^a^ | 1.29 ± 0.1^a^ | 2.14 ± 0.18^ghi^ | 2.23 ± 0.12^cde^ |
| **CA26** | 30.47 ± 1.43^c^ | 30.76 ± 1.79^defgh^ | 4.28 ± 0.32^a^ | 3.66 ± 0.33^a^ | 7.36 ± 0.4^abcdef^ | 5.15 ± 0.25^cdefghij^ | 1.76 ± 0.05^ab^ | 2.13 ± 0.15^a^ | 1.87 ± 0.04^bcd^ | 1.22 ± 0.31^a^ | 2.35 ± 0.27^def^ | 2.64 ± 0.22^d^ | 1.29 ± 0.08^a^ | 0.82 ± 0.08^bcd^ | 6.51 ± 0.3^a^ | 3.59 ± 0^a^ |
| **CA27** | 17.07 ± 0.68^ijkl^ | 32.8 ± 0.59^cdef^ | 3.36 ± 0.15^bcd^ | 3.17 ± 0.35^abcd^ | 5.94 ± 0.53^defghi^ | 3.31 ± 0.31^l^ | 1.26 ± 0.15^cdef^ | 1.89 ± 0.21^abc^ | 1.51 ± 0.11^cdefg^ | 0.77 ± 0.06^cde^ | 4.91 ± 0.32^b^ | 0.93 ± 0.33^lmnopqr^ | 0.88 ± 0.04^cde^ | 0.84 ± 0.12^bcd^ | 2.6 ± 0.15^efg^ | 2.55 ± 0.13^bc^ |
| **CA28** | 13.13 ± 0.06^op^ | 19.35 ± 1.75^lmno^ | 3.75 ± 0.1^ab^ | 2.91 ± 0.26^bcde^ | 5.78 ± 0.5^efghi^ | 4.86 ± 0.1^fghijkl^ | 1.31 ± 0.26^bcdef^ | 1.61 ± 0.09^abcdef^ | 1.52 ± 0.09^cdefg^ | 0.7 ± 0.05^cdefgh^ | 1.03 ± 0.01^klmn^ | 1.41 ± 0.08^hijk^ | 1.28 ± 0.29^a^ | 1.21 ± 0.15^a^ | 2.76 ± 0.13^def^ | 2.56 ± 0.16^bc^ |
| **CA29** | 23.5 ± 0.34^de^ | 45.65 ± 1.52^a^ | 3.88 ± 0.14^ab^ | 3.75 ± 0.21^a^ | 6.64 ± 0.12^bcdefgh^ | 5.75 ± 0.39^bcdefgh^ | 2.05 ± 0.31^a^ | 2.01 ± 0.1^ab^ | 2.09 ± 0.26^a^ | 1.21 ± 0.25^a^ | 1.34 ± 0.01^ijk^ | 1.81 ± 0.25^g^ | 0.98 ± 0.15^bc^ | 1.33 ± 0.09^a^ | 3.92 ± 0.15^b^ | 2.45 ± 0.13^bcd^ |
| **CA30** | 17.72 ± 0.23^hijkl^ | 38.25 ± 2.01^bc^ | 3.5 ± 0.17^bc^ | 3.25 ± 0.18^abc^ | 6.1 ± 0.43^cdefghi^ | 5.08 ± 0.82^defghijk^ | 1.48 ± 0.12^bcd^ | 1.79 ± 0.21^abcd^ | 1.56 ± 0.11^cdef^ | 0.77 ± 0.01^cdef^ | 1.08 ± 0.05^klmn^ | 6.95 ± 0.08^a^ | 1.21 ± 0.11^ab^ | 0.89 ± 0.12^b^ | 3.02 ± 0.08^cde^ | 2.55 ± 0.18^bc^ |
| **CA31** | 12.12 ± 0.13^q^ | 35.38 ± 1.83^bcde^ | 3.72 ± 0.21^ab^ | 3.54 ± 0.27^ab^ | 5.91 ± 0.33^defghi^ | 3.92 ± 0.32^jkl^ | 1.67 ± 0.19^abc^ | 1.83 ± 0.13^abcd^ | 1.62 ± 0.12^cde^ | 1.09 ± 0.09^ab^ | 0.88 ± 0.17^lmn^ | 0.88 ± 0.07^lmnopqrs^ | 1.26 ± 0.18^a^ | 0.84 ± 0.11^bcd^ | 2.84 ± 0.19^def^ | 2.39 ± 0.26^bcd^ |
| **CA32** | 30.1 ± 0.62^c^ | 38.22 ± 0.74^bc^ | 3.36 ± 0.03^bcd^ | 1.93 ± 0.12^ghijkl^ | 7.48 ± 0.54^abcdef^ | 4.43 ± 0.62^hijkl^ | 1.75 ± 0.18^ab^ | 1.66 ± 0.03^abcdef^ | 1.62 ± 0.17^cde^ | 0.83 ± 0.04^bcd^ | 0.61 ± 0.01^n^ | 1.02 ± 0.02^lmnop^ | 0.92 ± 0.19^cd^ | 1.3 ± 0.02^a^ | 3.01 ± 0.05^cde^ | 2.42 ± 0.34^bcd^ |
| **CA33** | 25.11 ± 0.6^d^ | 36.14 ± 1.87^bcd^ | 3.77 ± 0.18^ab^ | 3.64 ± 0.11^a^ | 8.17 ± 0.21^ab^ | 7.32 ± 0.53^b^ | 1.54 ± 0.13^bcd^ | 1.98 ± 0.19^ab^ | 1.49 ± 0.19^cdefgh^ | 0.89 ± 0.13^bc^ | 2.77 ± 0.22^d^ | 1.76 ± 0.18^gh^ | 1.28 ± 0.01^a^ | 0.85 ± 0.02^bc^ | 3.45 ± 0.18^bc^ | 2.59 ± 0.14^bc^ |
| **CA34** | 18.45 ± 0.5^ghijk^ | 30.49 ± 2.77^defgh^ | 3.48 ± 0.26^bc^ | 1.74 ± 0.23^hijkl^ | 7.25 ± 0.59^abcdefg^ | 6.32 ± 0.45^bcdefg^ | 1.43 ± 0.27^bcde^ | 1.76 ± 0.21^abcde^ | 1.97 ± 0.08^bc^ | 0.74 ± 0.11^cdefg^ | 0.74 ± 0.05^mn^ | 0.69 ± 0.06^pqrst^ | 0.86 ± 0.07^cde^ | 1.31 ± 0.31^a^ | 2.09 ± 0.25^ghi^ | 1.96 ± 0.12^defg^ |
| **CA35** | 16.5 ± 0.26^jklm^ | 39.84 ± 0.29^b^ | 3.33 ± 0.26^bcd^ | 3.3 ± 0.21^abc^ | 7.15 ± 0.31^bcdefg^ | 5.94 ± 0.05^bcdefgh^ | 1.22 ± 0.15^cdef^ | 1.5 ± 0.17^bcdef^ | 1.35 ± 0.3^efghi^ | 0.77 ± 0.05^cdef^ | 4.68 ± 0.1^b^ | 3.39 ± 0.09^c^ | 0.79 ± 0.1^cde^ | 0.76 ± 0.09^bcde^ | 1.91 ± 0.21^hijk^ | 1.57 ± 0.09^ghij^ |

***Note:** Values are means ± standard error (SE); Within columns, SE followed by different superscript letters (a, b, c, & so on) indicates significant differences (*p*<0.05) using One-way ANOVA Duncan multiple range test (DMRT). AG – above-ground (aerial) part, BG – below-ground part

**Table S3** HPLC profile of phenolic compounds: retention time, λ max, LOD, and LOQ

| **Compounds Name** | **ʎ max (nm)** | **RT (min)** | **Linearity range (µg/mL)** | **LOD (****µg/mL)** | **LOQ (µg/mL)** | **Equation** | **R ^2^** | **Peak purity** | | **Intra-day variation s (; n=15)** | |
| --- | --- | --- | --- | --- | --- | --- | --- | --- | --- | --- | --- |
|  |  |  |  |  |  |  |  | **Peak angle** | **Peak threshold** | **Retention time (RSD%** | **Peak area (RSD%)** |
| Catechin | 220 | 4.07 | 10-50 | 0.63 | 1.92 | y = 39828x - 38445 | 0.999 | 0.193 | 0.246 | 0.110 | 0.584 |
| Phloridzin | 220 | 13.01 | 10-50 | 6.7 | 20.31 | y = 22282x - 18962 | 0.987 | 0.095 | 0.248 | 0.170 | 0.596 |
| Ascorbic acid | 254 | 3.08 | 10-50 | 1.27 | 3.86 | y = 23186x - 11441 | 0.999 | 1.998 | 0.223 | 0.054 | 0.70 |
| Naringin | 220 | 10.84 | 10-50 | 6.58 | 19.94 | y = 25646x - 19417 | 0.988 | 0.436 | 0.245 | 0.166 | 0.389 |
| *p*-hydroxybenzoic acid | 254 | 5.85 | 10-50 | 1.77 | 5.36 | y = 56628x - 66067 | 0.999 | 0.051 | 0.216 | 0.094 | 0.086 |
| *o*-coumaric acid | 272 | 12.71 | 10-50 | 1.91 | 5.78 | y = 59545x - 10095 | 0.999 | 0.102 | 0.211 | 0.093 | 0.155 |
| *p*-coumaric acid | 310 | 9.12 | 10-50 | 1.77 | 5.38 | y = 72213x - 13321 | 0.999 | 0.079 | 0.212 | 0.180 | 0.308 |
| Ferulic acid | 320 | 10.03 | 10-50 | 6.75 | 19.98 | y = 58139x - 43645 | 0.987 | 0.074 | 0.21 | 0.200 | 0.165 |
